# Bacterioplankton Community Composition Along Environmental Gradients in Lakes From Byers Peninsula (Maritime Antarctica) as Determined by Next-Generation Sequencing

**DOI:** 10.3389/fmicb.2019.00908

**Published:** 2019-04-30

**Authors:** Antonio Picazo, Carlos Rochera, Juan Antonio Villaescusa, Javier Miralles-Lorenzo, David Velázquez, Antonio Quesada, Antonio Camacho

**Affiliations:** ^1^Cavanilles Institute for Biodiversity and Evolutionary Biology, University of Valencia, Valencia, Spain; ^2^Departamento de Biología, Facultad de Ciencias, Universidad Autónoma de Madrid, Madrid, Spain

**Keywords:** next-generation sequencing, Byers Peninsula, bacterioplankton, Maritime Antarctic lakes, environmental gradients

## Abstract

This study comprises the first attempt to describe the planktonic bacterial communities of lakes from Byers Peninsula, one of the most significant limnological districts in the Maritime Antarctica, leveraging next-generation sequencing (NGS) technologies. For the survey, we selected 7 lakes covering the environmental gradient from inland to coastal lakes, some of them sampled both in surface and deep waters. Analysis provided just over 85,000 high quality sequences that were clustered into 864 unique Zero-radius Operational Taxonomic Units (ZOTUs) (i.e., 100% sequence similarity). Yet, several taxonomic uncertainties remained in the analysis likely suggesting the occurrence of local bacterial adaptations. The survey showed the dominance of the phyla Proteobacteria and Bacteroidetes. Among the former, the Gammaproteobacteria class, more specifically the order Betaproteobacteriales, was the dominant group, which seems to be a common trend in nutrient-limited Antarctic lakes. Most of the families and genera ubiquitously detected belonging to this class are indeed typical from ultra-oligotrophic environments, and commonly described as diazotrophs. On the other hand, among the members of the phylum Bacteroidetes, genera such as *Flavobacterium* were abundant in some of the shallowest lakes, thus demonstrating that also benthic and sediment-associated bacteria contributed to water bacterial assemblages. Ordination analyses sorted bacterial assemblages mainly based on the environmental gradients of nutrient availability and conductivity i.e., salinity. However, transient bacterial associations, that included the groups Clostridiaceae and Chloroflexi, also occurred as being forced by other drivers such as the influence of the nearby fauna and by the airborne microorganisms. As we intended, our NGS-based approach has provided a much greater resolution compared to the previous studies conducted in the area and confirmed to a large extent the previously obtained patterns, thus reinforcing the view of Byers as a hotspot of microbial biodiversity within Antarctica. This high microbial diversity allows the use of these aquatic ecosystems and their bacterial assemblages as sentinels for the monitoring of adaptive responses to climate change in this rapidly warming area.

## Introduction

Environmental conditions in Antarctica impose severe restrictions to life. Accordingly, lakes there show relatively simple food webs and are dominated by microorganisms (Vincent and Laybourn-Parry(eds), 2008). Interactions based on microbial pathways are then responsible for a large part of energy transfer in these ecosystems. Ecological dynamics may differ, however, depending on the Antarctic region considered. In the maritime Antarctica in particular, the phenology of the ice-cover of lakes is very responsive to the air temperature because, during summer, it is close to the water freezing point. The benthic compartment in these lakes has usually a main role (Quesada and Velázquez, 2012), but during the transition to the ice-free periods, the productivity in the water column is enhanced because the high availability of light and fluxes of allochthonous nutrients (López-Bueno et al., 2009; Rochera et al., 2010). During these periods, major changes are expected to occur in the composition of planktonic microbial assemblages. In a framework of global warning that affects severely to these ecosystems (Camacho et al., 2012), it arises as a need to assess to what extent these microbial communities are resilient to disturbances, and what are the major structuring forces (e.g., nutrients, dispersal, etc.).

As part of the Maritime Antarctica, the ice-free region of Byers Peninsula has been established, in the last decades, as a reference site for terrestrial ecological studies (Quesada et al., 2009), including the limnology of inland waters. In general, nutrients are in short supply in Byers’ lakes (Toro et al., 2007; Rochera et al., 2013a), at least for non-coastal lakes, and the heterotrophic metabolism is expected to exceed primary production as most of these lakes are externally subsidized. Under these circumstances, the characteristics of the surrounding catchment are important to determine allochthonous supplies of carbon that may sustain bacterial heterotrophic production. Additionally, the inputs of the airborne and fauna-related bacteria will vary depending of lake’s characteristics and location, which may even influence, in concert with other internal forces, the vertical distribution of the microbial assemblages in these lakes. In fact, fecal bacterial taxa from marine birds’ drops are frequently found in surface waters even in lakes separated several kilometers from the coast (Villaescusa et al., 2013), though these taxa are more abundant in coastal lakes highly impacted by elephant seals (Gil-Delgado et al., 2013) and penguins (Santamans et al., 2017). On the other hand, the bottom of the deepest lakes in the area (5–8 m) is covered by dense moss carpets (Toro et al., 2007), and this determines a sort of “summer biological stratification” in these thermally homogeneous lakes, causing strong differences in the composition and relative dominance in the bacterial assemblages between surface and bottom layers (Villaescusa et al., 2013).

There are different studies addressing the prokaryotic diversity in terrestrial sites from the maritime Antarctica, both in aquatic ecosystems (Pearce and Galand, 2008) and soils (Pearce et al., 2012; Wang et al., 2015; Santamans et al., 2017). These studies have unveiled a higher microbial diversity than expected, considering the supposedly extreme environmental conditions. These studies also highlight the role of several abiotic and biotic factors in structuring these metacommunities (Pearce and Galand, 2008). Studies on the bacterial communities of Byers’ lakes using molecular methods have been conducted so far involving culture-independent techniques such as denaturing gradient gel electrophoresis (Villaescusa et al., 2010), cloning-sequencing (Villaescusa et al., 2013), and CARD-FISH hybridization (Papale et al., 2017). These studies ascertained the major microbial constituents in lakes and outlined some ecological patterns, yet they did not allow to detect relatively low abundant microorganisms in the consortia or just focused on a single model ecosystem. Next generation sequencing (NGS) techniques allow to increase the throughput of these analyses by increasing coverage and sensitivity, however, the use of such molecular tools is still relatively emergent for the study of Antarctic sites. To partly address this shortcoming, the present study reports the first survey conducted using NGS techniques to explore diversity patterns of planktonic microbial communities in one of the most significant limnological districts of the Maritime Antarctica (Byers Peninsula). As a further point of interest, this study will allow us to contrast the outcomes and patterns revealed by previous studies conducted so far in the site with those resulting from this deepest sequencing approach.

## Materials and Methods

### Study Site

**FIGURE 1:**
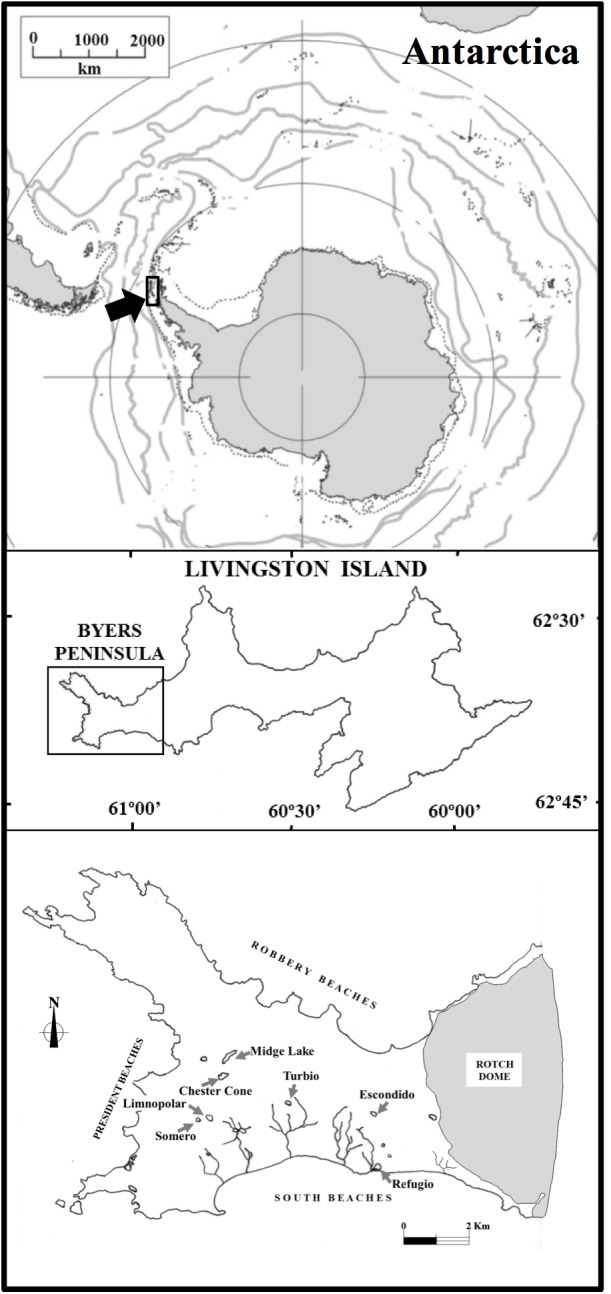
Map showing lake locations on Byers Peninsula within the Antarctic continent and the lakes studied.

Byers Peninsula is located at the west side of Livingston Island (Figure 1; 62°04′35″–62°40′35″S, 60°54′14″–61°13′07″W). The site is one of the largest ice-free areas in the Antarctic Peninsula region, with 60 km^2^ of ice-free area during the summer. Byers Peninsula presents a complex drainage network, with many lakes, ponds, streams and wetlands covering part of the land surface (Figure 1). These freshwater ecosystems are distributed in two main geomorphological areas (Toro et al., 2007; Rochera et al., 2013a). One of them entails extensive raised marine platforms and beaches bordering the coast, whereas the wider area comprises an elevated platform which lay between 85 and 100 m.a.s.l. This upland is plentiful of erosive and depositional features of glacial origin. The weather in Byers Peninsula is representative of the maritime Antarctic region and contrast greatly with the climate conditions of the continental part. It is characterized by cloudy skies, windy conditions and high precipitation, but milder temperatures than in the continent (Rochera et al., 2010).

Among the studied lakes (Table 1), those from the plateau (lakes Chester Cone, Midge, Escondido, Limnopolar, Turbio and Somero) occupy a landform modified by fluvial and periglacial processes that favor water retention. Some of them, such as Chester Cone Lake, are located in small catchments near or bounded by isolated volcanic plugs. In general, these lakes show clear surface outlets, except in the case of Lake Escondido, which is located between basaltic hills. Most inland lakes, especially Chester Cone and Midge showing the widest catchments, have a low percentage of vegetation coverage in these catchments (Table 1). Other parts of the plateau show, instead, a higher, thought modest, vegetation coverage, like those occupied by the smaller catchments of lakes Limnopolar and Somero. In clear contrast with the plateau, the coastal area where the Lake Refugio is located, is largely covered by moss cushions.

**Table 1:** Geographical location and main characteristics of the studied lakes from Byers Peninsula.

Lake	X-UTM	Y-UTM	Conductivity (μS/cm)	Catchment size (km^2^)	Lake Surface (km^2^)	Maximum depth (m)	% Vegetation	TP (μM)	Chl-a (μg l^−1^)
Chester Cone	597500	3053550	58	0.09	0.039	5.0	2	0.28	0.07
Midge Lake	597700	3054150	69	0.27	0.054	8.2	2	0.23	0.13
Somero	596800	3052150	81	0.06	0.011	0.5	4	1.02	0.94
Turbio	598000	3051800	60	0.58	0.021	7.8	1	1.27	0.37
Limnopolar	597100	3052200	66	0.58	0.023	5.5	4	0.34	0.10
Escondido	599475	3052650	60	0.08	0.022	4.5	1	0.28	0.38
Refugio	602200	3050550	148	0.12	0.016	0.5	20	16.84	23.41

### Water Sampling and Assessment of Trophic Status Metrics

Sampling for this survey was conducted during the austral summer 2007–2008, at the ice-free period (January–February), in lakes Chester Cone, Midge Lake, Escondido, Limnopolar, Turbio, Somero, and Refugio. During these months, lakes from Byers Peninsula usually show a thermal homogeneity and a (thermally) unstratified water column. Samples in all lakes were obtained from surface depths (0.5–1 m). To assess the unequal distribution of microorganisms through the water column in lakes showing higher vertical heterogeneity (Villaescusa et al., 2013), additional samples were obtained in lakes Limnopolar and Chester Cone at depths near the bottom, namely 3.5 and 4.5 m, respectively, on the top part of the aquatic moss carpets. Water temperature, conductivity and pH were measured with a Yellow Springs Instrument (YSI) Water Logger System multiprobe model 556 MPS. In lakes deeper than 1 m, Limnopolar, Chester Cone, Turbio, Escondido and Midge, water samples were taken with a 5 L Kemmerer bottle. For shallower lakes, such as Lake Somero and Lake Refugio, samples were taken directly. All lakes were sampled approximately in the center of the lake. Samples for DNA analyses were then filtered with a vacuum system onto 0.2 μm polycarbonate filters (Nucleopore, Whatman), then, filters were stored frozen (−20°C) in Allprotect Tissue Reagent (QIAGEN) until analysis. The database to show Supplementary Data on the trophic status and bacterial abundances of lakes was made by compiling previously published data from surveys conducted in these lakes during different austral summers, from 2001/2002 to 2008/2009 (Toro et al., 2007; Rochera et al., 2010, 2013a; Villaescusa et al., 2013; Papale et al., 2017). Chlorophyll-a (Chl-*a*) and total phosphorus (TP) concentrations were used as indicators of the trophic status of lakes. Methods are detailed in the above mentioned references.

### DNA Extraction, Illumina 16S Sequencing and Taxonomic Classification

DNA extraction from each filter was performed with the EZNA Soil DNA isolation kit (Omega Bio-Tek, Inc., Norcross, GA, United States) following the instructions given by the supplier. Sequencing of the region V4 of the 16S rRNA gene was done using the Illumina MiSeq system (2×250 bp) at the genomics facilities of the Research Technology Support Facility of the Michigan State University, United States. For each sample, Illumina compatible, dual indexed amplicon libraries of the 16S-V4 rRNA hypervariable region were created with primers 515f/806r. PCR reactions are composed of 5 μL of 4 μM equimolar primer set, 0.15 μL of AccuPrime Taq DNA High Fidelity Polymerase, 2 μL of 10× AccuPrime PCR Buffer II (Thermo Fisher Scientific, catalog no. 12346094), 11.85 μL of PCR-grade water, and 1 μL of DNA template. The PCR conditions used consisted of 2 min at 95°C, followed by 30 cycles of 95°C for 20 s, 55°C for 15 s, and 72°C for 5 min, followed by 72°C for 10 min (Kozich et al., 2013). Completed libraries were batch normalized using Invitrogen SequalPrep DNA Normalization Plates. Then, the product recovered from the plates was pooled. The pool was QC’d and quantified using a combination of Qubit dsDNA HS, Agilent 4200 TapeStation High Sensitivity DNA and Kapa Illumina Library Quantification qPCR assays. This pool was loaded on a standard Illumina MiSeq v2 flow cell and sequencing was performed in a 2×250 bp paired end format using a MiSeq v2 500 cycle reagent cartridge. Custom sequencing and index primers complementary to the 515/806 target sequences were added to appropriate wells of reagent cartridge. Base calling was done by Illumina Real Time Analysis (RTA) v1.18.54 and output of RTA was demultiplexed and converted to FastQ format with Illumina Bcl2fastq v2.19.1. Sequences were processed using the UPARSE pipeline using USEARCH v11.0.667 (Edgar, 2013). After merging of read pairs, the dataset was filtered by a maximum number of expected errors of 0.5. Chimeric sequences were removed with USEARCH v11.0.667 utilizing the UCHIME (Edgar, 2013), against the SILVA 128 database. Filtered sequences were clustered in Zero-radius Operational Taxonomic Units (ZOTUs), which are sequences with 100% of identity. Taxonomic assignment was done with SINA v1.2.1152 using SILVA 128 database. SINA uses Lowest Common Ancestor method (LCA). We configurated a “Min identity” of 0.7 and a maximum number of search results of 1 per sequence results in “best match” type. Sequences with low alignment quality (<75%) and sequences identified as mitochondria or chloroplasts were removed from the analysis. Original ZOTU table were normalized by rarefying the reads of all samples to the minimum thresholds of 2000, 4000, and 10000 reads/sample. Rarefactions were repeated 100 times to avoid the loss of less abundant ZOTUs, and the rarefactions were unified in three average rarefied ZOTU tables (with thresholds of 2000, 4000, and 10000 reads/sample) A Mantel test was performed with the three ZOTU tables, showing no significant differences among them. Therefore, in order to avoid the loss of samples with lower number of reads, further analyses were done based on the ZOTU table with the threshold of 2000 reads/sample. Quality reads were additionally clustered into OTUs (Operational Taxonomic Unit) with a 97% identity level by USEARCH (v11.0.667) (Edgar, 2013). OTU representative sequences were classified and processed in the same way as ZOTUs. ZOTUs are OTUs with a similarity of 100% instead of 97%, the UNOISE2 algorithm used allows a better PCR correction in Illumina amplicons reads (Edgar, 2016).

All sequence data from this study have been deposited in the Sequence Read Archive (SRA) of the National Center for Biotechnology Information (NCBI), BioProject accession number PRJNA528697. Krona charts were built with normalized ZOTU table (Ondov et al., 2011), the interactive Krona charts for all samples are available as Supplementary Material).

To estimate the diversity of the samples, the ZOTU, classified at 100% sequence similarity were processed using the statistical software PAST V3.0 (Hammer et al., 2001). Then, diversity (Shannon diversity index), evenness (Shannon evenness index) calculated according to Hill (1973), Chao1 of Illumina reads, and richness, were determined.

### Multivariate Ordination Analyses

To analyze the influence of environmental gradients on planktonic bacterial communities in surface water samples, Cluster analysis (HeatMap), non-metric multidimensional scaling (NMDS) ordination, as well as Redundancy analysis (RDA), were performed at family level based on the Bray–Curtis dissimilarity between bacterial surface communities for each lake. HeatMap and NMDS were also performed further to analyze the influence of vertical structure of the planktonic bacterial assemblages in the water column by comparing the samples of surface and deep waters in lakes Limnopolar and Chester Cone.

HeatMap workflows was carried out using “pheatmap” package (version 1.0.12). NMDS ordinations were constructed using the vegan package version 2.5–4 (Oksanen et al., 2016) in the R programming environment (R Development Core Team, 2015) to describe community dissimilarity in unconstrained space. ZOTU tables were Hellinger transformed to calculate ordination based on Hellinger distances (as recommended by Legendre and Gallagher, 2001). RDA models were also built in the vegan package in order to describe the planktonic community structure ordinations in the environmentally constrained space.

For the RDA, a matrix of environmental variables has been used. This comprises 7 variables for the 7 studied lakes (Table 1). The environmental variables used were the catchment area, lake surface area, lake depth, percentage of the lake catchment covered by vegetation (mainly lichens and mosses but, for coastal areas, also vascular plants such as *Deschampsia antarctica and Colobanthus quitensis*), water conductivity, and the historical data of Chl-*a* and TP concentrations in water. For NMDS and RDA analyses the taxa matrix at family level is composed by the total reads (normalized by rarefaction to 2000 reads/sample) for each lake. For HeatMap analysis at the family level the taxa matrix is composed by the reads (normalized by rarefaction to 2000 reads/sample) of most abundant families that account for percentages above 1% of the total reads at least in one of the lakes.

## Results

### Environmental Characteristics and Trophic State of Lakes

**FIGURE 2:**
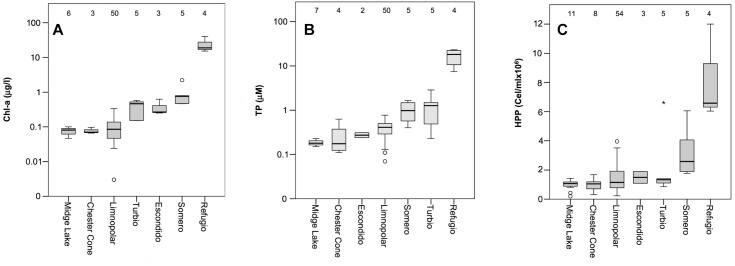
Box plot of historical data of concentrations of **(A)** chlorophyll-*a* (Chl-*a* in log-scale), **(B)** total phosphorus (TP in log-scale), and **(C)** the abundance of heterotrophic bacterioplankton (HHP) measured in the lakes studied. Numbers inside the charts indicate the number of samples. The boundaries of the box indicate the 25th and 75th percentiles. Solid line indicates median value. Lakes in each plot are ordered from lowest to highest median value of the variable reported in the chart.

Lakes were almost thawed during sampling. Only a small part of the icecap remained in some lakes from the plateau for just a part of the summer. The water electrical conductivity measured in lakes during the 2007–2008 sampling season was low, and varied between 57 and 203 μS cm^−1^, being higher in the coastal lake Refugio, all this agreeing with historical data showing higher conductivities in lakes located closer to the sea. Chl-a and TP concentrations, obtained from historical data, and used to estimate trophic status, varied widely among the studied lakes (Figure 2). In the plateau lakes (i.e., all except Refugio), total phosphorus (TP) concentrations ranged from nearly undetectable levels (<0.03 μM) to around 3 μM (Figure 2). Among these lakes, Chester Cone and Midge showed the lowest TP and Chl-a. The shallowest lake from the plateau, Lake Somero, with a higher relative relevance of benthic communities, showed higher TP concentrations. In any case, the highest TP concentrations (average 16.84 ± 7.10 μM) were consistently observed in the coastal Lake Refugio. The pattern of Chl-a concentrations mimicked that of TP, with lower values (average 0.08 ± 0.02 μg l^−1^) in the deeper ultra-oligotrophic lakes from the central plateau, contrasting with the much higher concentrations (23.41 ± 11.50 μg l^−1^) in the coastal Lake Refugio (Figure 2). Regarding bacterioplankton abundance, historical surveys conducted in these lakes showed increasing abundances from inland to coastal sites following a trophic gradient (Figure 2), though the shallower lake of the plateau, Lake Somero, also showed higher bacterial abundance as occurred for TP and Chl-a concentration. Overall, bacterial numbers ranged two orders of magnitude between minimum and maximum values historically found, from 2.01 × 10^5^ to 1.20 × 10^7^, when comparing the most oligotrophic lakes to the richer coastal Lake Refugio.

### Planktonic Prokaryotic Assemblages and Diversity in Surface Waters

**FIGURE 3:**
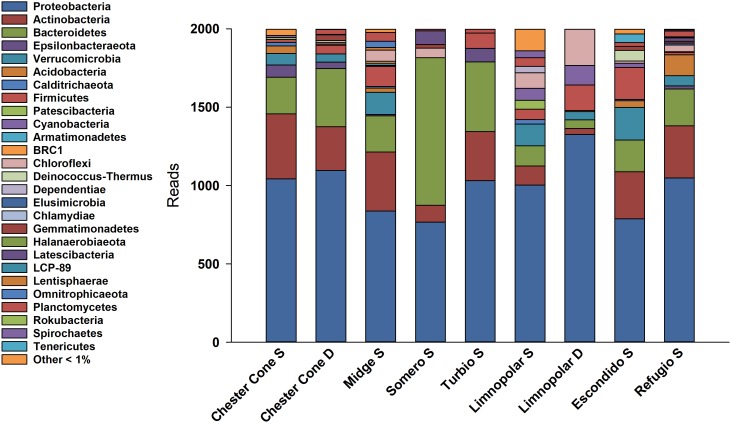
Relative percentages of reads assigned to phyla in the surface water samples of the studied lakes, obtained from massive sequencing analysis over PCR amplicons of the V4 region of 16S rRNA.

**FIGURE 4:**
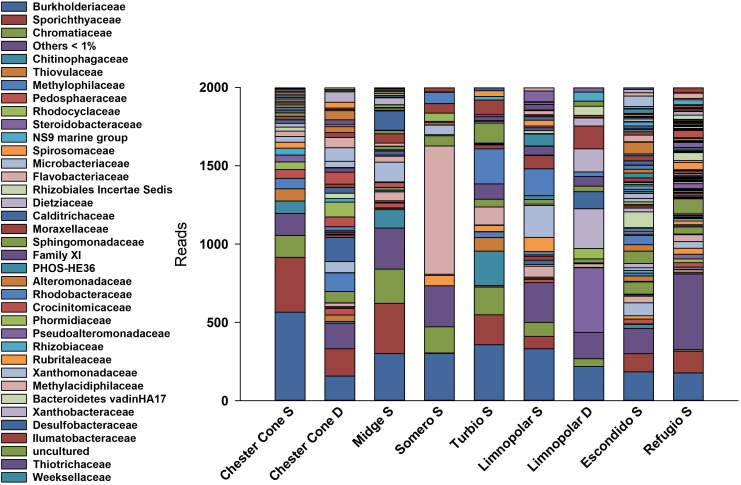
Relative percentages of reads assigned to families in the surface water samples of the studied lakes, obtained from massive sequencing analysis over PCR amplicons of the V4 region of 16S gene.

**FIGURE 5:**
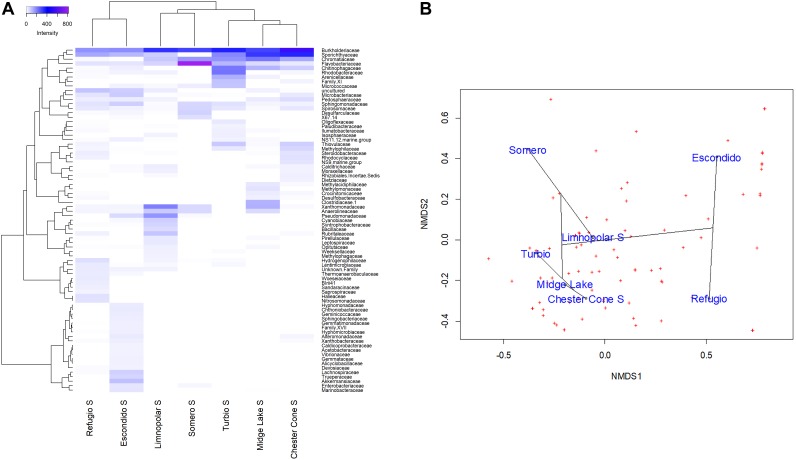
**(A)** Heatmap of two-way cluster analysis performed on the bacterioplankton community composition at family level (families with at least one sample with more than 1% in relative abundance) in the surface waters of the studied lakes. Both the taxa and the samples were clustered using Bray–Curtis dissimilarities. The color intensity in the cluster dendrogram correspond to the abundance of normalized reads. **(B)** A non-metric multidimensional scaling (NMDS) plot based on Bray–Curtis dissimilarity of bacterial taxonomic composition at family level (all families) across the studied lakes.

A total of 85809 high quality sequences were obtained from the whole set of samples from the different lakes and depths studied. All these sequences were clustered into 864 unique Zero-radius Operational Taxonomic Units (ZOTUs) with a 100% sequence similarity. Its taxonomic assignations at the different taxonomic levels are detailed in the interactive Krona charts (Supplementary Figure S1). The metagenomic data analyses were performed both in ZOTUs (Figure 3, 4) and 97% OTUs (data not shown). The general patterns of relative and absolute abundance at the different taxonomic studied levels were similar using both approaches. However, the percentage of unclassified taxa was lower or at least equal, depending on the lakes, in ZOTUs clustering compared to the 97% OTUs. Actually, in some cases, the number of unclassified taxa in OTUs increased up to twice such as in the surface samples of lakes Turbio and Limnopolar. These variations gave only a slight change in the cluster analysis (Figure 5A), causing Lake Limnopolar clustering with the group formed by Refugio and Escondido, whereas all the rest of clustering kept the same (data not shown).

The ZOTUs richness in the studied lakes ranged from 49 to 425, being the lowest in Lake Somero and the highest in Lake Refugio (Table 2). Lakes Refugio and Escondido showed the highest values of the Shannon Diversity and Chao1, whereas, when only surface samples were considered, lakes Turbio and Somero showed the lowest values for these parameters (Table 2).

**Table 2:** Summary of diversity parameters obtained from the Illumina sequencing analysis in the studied lakes from Byers Peninsula.

	Chester S	Chester D	Midge	Somero	Turbio	Limnopolar S	Limnopolar D	Escondido	Refugio
**Richness**	100	122	129	49	52	75	52	206	425
**Shannon Index**	3.737	4.152	3.927	3.171	3.193	3.764	3.162	4.871	5.461
**Evenness**	0.420	0.521	0.393	0.486	0.468	0.575	0.454	0.633	0.554
**Chao-1**	100	122	129	49	52	75	52	207	425

*For lakes sampled in different layers S means surface sample, and D means deep sample.*

The resulting 16S rRNA amplicons were classified into 27 bacterial phyla (Figure 3). In average, for surface samples of all studied lakes, Proteobacteria represented the higher percentage (47.3%) followed by Bacteroidetes (17.4%), Actinobacteria (14.3%) and Verrucomicrobia (4.6%). Among the classes of Proteobacteria, Gammaproteobacteria (including the former Betaproteobacteria Class, now within the Gammaproteobacteria in SILVA, Parks et al., 2018) and Alphaproteobacteria were the most abundant, with average frequencies of 37.4 and 6.4%, respectively. Within the Gammaproteobacteria class, the order of Betaproteobacteriales (formerly within the Class Betaproteobacteria) was the most abundant with 18.8%.

Noticeably, the highest percentage of Bacteroidetes (∼47.1%) was found in Lake Somero, linked to the conspicuous occurrence (40.1%) of the family Flavobacteriaceae (Figure 4) with the dominant genera *Pseudarcicella* and, particularly, *Flavobacterium*. By contrast, Proteobacteria showed the lowest dominance (38.3%) in this lake when compared to the others. On the other hand, the widespread dominance of order Betaproteobacteriales was mainly due to the dominance (15.1%) of the ubiquitous family Burkholderiaceae (Figure 4), with genera such as *Polaromonas, Polynucleobacter, Rhodoferax* and *Limnohabitans* (Supplementary Figure S1). The relative abundance of *Limnohabitans* was usually higher in lakes from the plateau, showing coverages between 3 and 8%. On the other hand, *Polynucleobacter* was also very abundant, with maximum occurrence of nearly 14% in the surface waters of Chester Cone (Chester Cone S). The genus *Polaromonas* also showed a ubiquitous distribution, although it was somewhat higher in lakes Midge and Somero, with similar percentages averaging 10%.

The segregation of families among the surface samples of lakes is shown in the heatmap of Figure 5A. The most ubiquitous, and generally more abundant, families were Burkholderiaceae, Sporichthyaceae and Sphingomonadaceae. Nevertheless, each lake also displayed some families more specifically. The analysis revealed three main clusters according to the differences on the families composition. The first one consisted of inland lakes, including some of the deepest, Chester Cone, Midge and Turbio, whereas a second one was arranged by the adjacent lakes Limnopolar and Somero. Finally, lakes Escondido and Refugio, composed the third cluster. This clustering is also visualized in the NMDS analysis (Figure 5B). The first group composed by three inland lakes Chester Cone, Midge and Turbio was particularly related with the occurrence of methylotrophs such as Methylophilaceae, Methylomonaceae, but also the families Desulfobacteraceae, Thiovulaceae, Rhodobacteraceae and Clostridiaceae. The other inland lakes differed in the identity of their determining families. Thus, Rubritaleaceae, Cyanobiaceae, Syntrophobacteraceae, Bacillaceae or Leptospiraceae were characteristics of Lake Limnopolar, whereas families determining more the assemblage of Lake Somero were Flavobacteriaceae, Desulfarculaceae, Spirosomaceae and Sphingomonadaceae. In the case of Lake Escondido, the distinguishing families were Akkermansiaceae, Lachnospiraceae, Trueperaceae, Marinobacteraceae and Alteromonadaceae, and for Lake Refugio these were Hydrogenophilaceae, Halieaceae, Nitrosomonadaceae, Woeseiaceae and Thermoanaerobaculaceae.

### Environmental Gradients and Their Relationship With Surface Water Sequence Data (Canonical Ordination Modeling)

**FIGURE 6:**
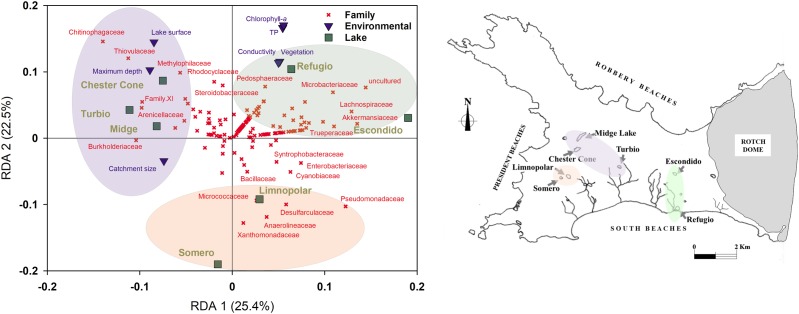
(Left) Redundancy Analysis (RDA) triplot between environmental variables (catchment area, lake surface area, lake depth, percentage of the lake catchment covered by vegetation, water electrical conductivity, TP, and Chl-*a*) and the bacterioplankton community composition at family level; and (Right) Geographic location of lakes in the Byers Peninsula colored as for the RDA groups.

An RDA was performed with environmental data and taxonomic affiliations within the bacterial assemblages at family level of surface samples of all studied lakes (Figure 6). The variance explained was 25.4 and 22.5% for the first and second axes, respectively. In this case, lakes Refugio and Escondido were positioned in the second quadrant of the RDA (i.e., positive sides of both axes, respectively) which was mainly associated to the occurrence of families such as Pedosphaeraceae, Microbacteriaceae, Lachnospiraceae and Akkmansiaceae. However, the position of Lake Refugio was more determined by its higher trophic status, as it was associated to Chl-*a* and TP concentrations. In opposition, the samples from some of the sites more oligotrophic and less prone to receive external inputs (i.e., more isolated lakes such Midge and Chester Cone) were situated in the negative part of the first axis of the RDA, which were linked to families with less pervasive members. On the other hand, the nearby lakes Limnopolar and Somero, although they differed in their trophic status, were positioned both in the negative side of the second axis, sharing a major presence in their assemblages of families such as Xanthomonadaceae, Micrococcaceae and Desulfarculaceae. It should be noted that a stream connects Lake Somero to the downstream Lake Limnopolar.

### Vertical Structure of the Planktonic Community in the Water Column of Lakes Chester Cone and Limnopolar

**FIGURE 7:**
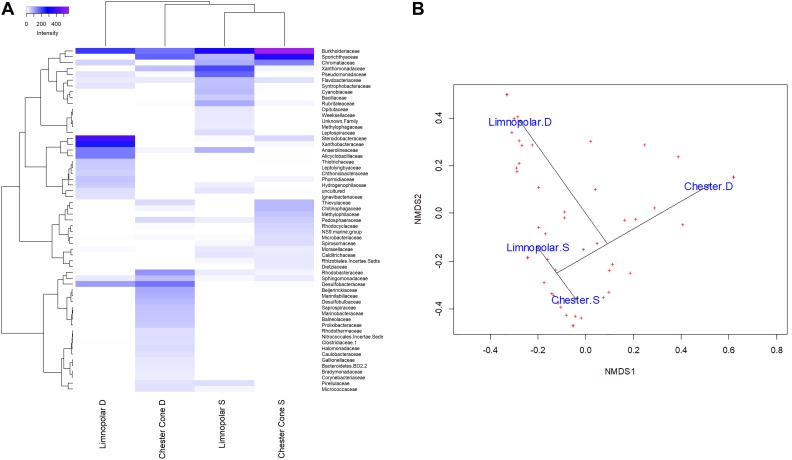
**(A)** Heatmap of two-way cluster analysis performed on bacterioplankton community composition at family level (families with at least one sample with more than 1% in relative abundance) in the surface and deep water samples of lakes Limnopolar and Chester Cone. Both the taxa and the samples were clustered using Bray–Curtis dissimilarities. The color intensity in the cluster dendrogram correspond to the abundance of normalized reads. **(B)** A non-metric multidimensional scaling (NMDS) plot based on Bray–Curtis dissimilarity of bacterial taxonomic composition at family level (all families) in the surface and deep water of lakes Limnopolar and Chester Cone.

The vertical distribution of the bacterial community was assessed in lakes Limnopolar and Chester (Figure 7). No clear pattern was found on ZOTUs richness between depths (Table 2), as it was somewhat higher in the upper layer of Lake Limnopolar compared to the bottom, whereas in the Chester Cone Lake, where overall richness was almost double, the vertical pattern seemed to be the opposite. In both lakes, abundant families including cosmopolitan member such as Burkholderiaceae and Sporichthyaceae were rather uniformly distributed through the water column, but the distribution of other groups changed markedly with depth. Thus, in Lake Limnopolar, the families Xanthomonadaceae and Pseudomonadaceae were more abundant in the upper layer, whereas Steroidobacteraceae and Xanthobacteraceae concentrated in the bottom. Other families such as Alicyclobacillaceae, Thiotrichaceae, as well the cyanobacterial Leptolyngbyaceae and Phormidiaceae, also occurred particularly in the deeper layer of this lake, the latter probably associated to the benthic mosses. In addition, Proteobacterial families related to the sulfur cycle (i.e., Desulfobacteraceae and Desulfobulbaceae) were mainly retrieved from the bottom samples in both lakes, particularly in Lake Chester Cone. Also, in this lake, members of some families such as Sphingomonadaceae, Marinobacteraceae, Caulobacteraceae and Gallionellaceae dominated also in relative terms in the deepest sample. On the other hand, it was striking the occurrence in these two lakes of some anoxygenic microorganisms such as purple sulfur bacteria (Chromatiaceae) and Anaerolineaceae, the later belonging to the Chloroflexi phylum, which can also be associated to the specific conditions created within the moss carpets in the bottom samples.

## Discussion

Lakes surveyed in Byers Peninsula include a variety of morphological features and trophic status ranging from ultra-oligotrophic to eutrophic. In general, most lakes from the central plateau are medium-depth or shallow lakes with small watersheds and mostly ultra-oligotrophic, whereas those from the coastal area are shallow and experience higher nutrient enrichment because of the activity of marine fauna (mammals and birds) in their surroundings, especially the southern elephant seal *Mirounga leonina* (Gil-Delgado et al., 2013) in South Beaches. Furthermore, the exposure to sea spray provides an additional supplement of nutrients to all lakes, which is higher in coastal sites (Rochera et al., 2013a). Actually, the water of Byers’ lakes shows a Na/Cl ratio near to that of the sea (Toro et al., 2007), as well as a sodium enrichment relative to calcium higher than the expected by the rock weathering, which is otherwise quite active in the region (Lyons et al., 2013). This is explained by the overall maritime influence in the hydrochemistry of the area.

We decided to perform our sequence analysis with denoised OTUs, which provides ZOTUs rather than the classical OTUs at 97% identity threshold. This resolves better the intra-taxa variation in different clusters. This is because an species can be divided into several ZOTUs, whereas one OTUs may otherwise include several species. Certainly, most of the metagenomics studies in extreme environments done up until now have been conducted using OTUs, however, we found that differences between both methods are minor, thereby our results are comparable to other diversity studies carried out in similar environments. Our NGS (Illumina Miseq) analysis over PCR amplicons of microbial communities based on ZOTUs shows the dominance of order Betaproteobacteriales and the *Flavobacterium* group of the Bacteroidetes, which matches with the main results previously retrieved by Papale et al. (2017) for samples from Lake Limnopolar using CARD-FISH. However, in our work, a significant proportion of ZOTUs (25%) could not yet be assigned to any genera, which likely suggest an important presence of yet undescribed taxa that could represent locally adapted bacterial groups. To simplify the outcomes of our ordination analysis and the interpretation of results, we went deeper only with ZOTUs that could be assigned to already described taxa. Despite these taxonomic gaps and attending to global diversity parameters (Table 2), our findings provide a valuable insight into the diversity of prokaryotes found in Maritime Antarctic lakes.

Although most of the differential ecological features are often found at the family level or below (Tamames et al., 2010), also highest taxonomic levels, even phyla composition within the community, can show among their components some ecological features of the environment. As such, the dominance of Betaproteobacteriales in freshwater bacterial communities seems to be a general trend in Antarctica (Pearce and Galand, 2008 and cites therein; Valdespino-Castillo et al., 2018), which could be related with the widespread adaptations to nutrients scarcity in most members of this group. Accordingly, some Proteobacterial taxa reported from our samples are typical of ultraoligotrophic environments, such as *Bradyrhizobium* and several genera of the family Burkholderiaceae (e.g., *Polaromonas*, *Delftia*, *Rhodoferax*, *Hydrogenophaga* and *Limnohabitans*), all of them described as diazotrophic inhabitants of Antarctic sites (Niederberger et al., 2012; Nash et al., 2018). *Polaromonas* for instance, is quite abundant on glacier surfaces of polar habitats and can be transported across large distances from a variety of different environments (Gawor et al., 2016). This genus is also known to establish symbiotic relationships with phototrophic organisms (Kanzler et al., 2005), which increase its adaptive capacity likely favoring its ubiquity in cold environments. Noticeably, a subcluster from to the genus *Polynucleobacter* (Betaproteobacteriales), has been reported as endemic from Antarctica, showing both genetic and phenotypic features that differenciate this endemic clade from other located within other geographical locations (Hahn et al., 2015).

In lakes with higher nutrient availability, such as Lake Turbio and, especially, Lake Refugio, the above-mentioned diazotrophic taxa presented much lower percentages, thus suggesting that trophic status plays a major role in determining the composition of microbial assemblages also in Maritime Antarctic lakes. However, there may be other determining external factors. For instance, in Lake Refugio, which shows the highest values of the Shannon index, the feces of the surrounding elephant seal colonies are washed into the lake, but also penguins can frequently be observed nearby. These inputs, additionally to the proximity of the sea, are expected to strongly influence the bacterial community composition of this site, otherwise increasing diversity with sustained inputs of allochthonous bacteria that increase the diversity over of local taxa. The abundance of Chloroflexi in this eutrophic coastal lake matches well with the observed in previous studies in Byers, even in those focused in the sediments (Gugliandolo et al., 2016). This phylum has been found in microbial communities from sites strongly impacted by penguin activity causing high guano inputs, in both soils and sediments from different Antarctic locations (Zhu et al.,2015; Santamans et al., 2017).

Our data show increasing bacterial numbers from inland to coastal sites following a trophic gradient. In addition, not only the abundance but also the activity of this bacterioplankton has been previously described to follow the same pattern (Villaescusa et al., 2010). This is also because lakes located in the plateau are comparatively more isolated than the coastal ones, although not totally, from external factors. Medium depth lakes from the plateau show a cold monomictic thermal regime, with an inverse stratification in winter and a seasonal circulation during summer. Accordingly, a notable homogeneity of the water column concerning biotic components would be expected during the ice-free periods as that of our study, thus surface and deep samples should likely show similar bacterial assemblages. However, there are external drivers such as the waterborne transport from the catchment and sea sprays that selectively provides microbial taxa to surface waters (Villaescusa et al., 2013), which can explain why we observed different microbial assemblages in the vertical profile of lakes sampled at different depths. Additionally, the presence of dense (more than 1 meter thick) moss carpets in the bottom of the deepest plateau lakes, creates a stable and differential environment that provides niches for bacterial taxa that cannot thrive in the more homogeneous surface layers. Thus, in samples taken from the bottom of lakes Chester Cone and Limnopolar we found assemblages differentially dominated by filamentous cyanobacteria or by other bacterial families related to the sulfur cycle (i.e., Desulfobacteraceae and Desulfobulbaceae), more commonly related to benthic environments. These assemblages from the deep layers, particularly in the case of Chester Cone Lake, are also well represented by *Sphingomonas* (Sphingomonadaceae), *Marinobacter* (Marinobacteraceae), *Brevundimonas* (Caulobacteraceae), *Gallionella* (Gallionellaceae) and *Hydrogenophaga* (Burkholderiaceae), which have been described in pristine environments from the maritime Antarctica (Pearce et al., 2012), and are unrelated with impacted freshwater sites.

A remarkable finding was the occurrence, in bottom samples from the deeper lakes of the plateau, of anoxygenic microorganisms such as Anaerolineaceae and purple sulfur bacteria. The former has been described forming consortia with methanogens involved in the anaerobic degradation of alkanes (Liang et al., 2016). On the other hand, purple sulfur bacteria are mostly inactive under aerobic conditions as oxygen suppresses the synthesis of bacteriochlorophyll, and they lack electron donors for photosynthesis under such conditions. These taxa could be present incidentally in the sample. They likely belong to winter-based communities occurring in the bottom of the lakes when they are covered by ice and inversely stratified (Rochera et al., 2010), which facilitates that an anoxic layer develops close to the bottom. On this sense, this would represent an analogy with that observed in Antarctic lakes permanently frozen Antarctic lakes (Karr et al., 2003). Once the lake’s ice thaws and then the water column is mixed, these anaerobic microorganisms likely remain, although inactive, for some period within the entire water column. On this sense, it should be noted that our sampling was conducted when the complete melt-out had not yet occurred in some of these lakes, thereby suggesting that the transition to the whole oxygenated water column must have been very recent.

Some of the variability observed in bacterial assemblages may otherwise respond to landscape characteristics. *Flavobacterium* in particular, which has been described as closely associated to Antarctic cyanobacterial mats by several studies (McCammon and Bowman, 2000; Bernardet and Bowman, 2006), was very abundant in Lake Somero. This shallow lake, in comparison to the others, is almost totally surrounded by cyanobacterial microbial mats, which also cover part of the lake bottom (Fernández-Valiente et al., 2007; Toro et al., 2007; Rochera et al., 2013b). In very shallow lakes, such as Lake Somero, there is a wave-induced removal of sediments favoring nutrients turnover, but also retrieving sediment-associated bacteria. This is additionally favored by the burrowing activity of the abundant fairy shrimps *Branchinecta gaini* (Toro et al., 2007), which are very abundant in Lake Somero. The relevance of sediment suspension into waters of shallow lakes is also supported by the high amounts of pheopigments relative to the total algal biomass observed in the shallowest lakes in the site (Rochera et al., 2013a). Also, *Pseudarcicella* (Spirosomaceae), which is a genus commonly linked to nutrient-rich sites (Li et al., 2017), appeared in the bacterial community of Lake Somero (3.4%), Turbio (2%) and Refugio (1.9%), favored by the nutrient inputs linked to sediment suspension, for lakes Somero and Turbio, or to external sources, as in Lake Refugio.

Regarding the community composition along environmental gradients, these bacterial communities are expected to be far from equilibrium as they are primarily controlled by environmental factors, which involves a low buffering capability against external impacts. This would explain that the ordination of the surface samples of lakes produced by the RDA (Figure 6) relates to their geographical proximity, revealing the major influence of the nearby fauna and airborne microorganisms in structuring these microbial assemblages. It should be noted at this point that the fauna distribution and the strength of the sea spray potentially ejecting microorganisms are not the same among different locations within the Peninsula Byers. This also means that the dynamics and distribution along gradients of these communities may not be totally predictable, though being sensitive to changes in environmental parameters such as the nutrient availability.

As an example of the influence of the nearby fauna, Santamans et al. (2017) reported relatively high abundances of bacteria belonging to the phylum Firmicutes, and more specifically to the order Clostridiales and Bacillales, in soils affected by penguin depositions. The order Clostridiales appeared in the coastal Lake Refugio, which is frequented by marine animals such as penguins and, especially, elephant seals. Additionally, this order was also detected in Lake Turbio, whereas members of the order Bacillales appeared in lakes Limnopolar and Escondido, though showing low coverages. Despite the relative relevance of these taxa in lake waters is much lower than in soils from animal colonies, this indicates that, even in inland lakes, there is some influence of fecal drops of marine animals, mainly marine birds as skuas and terns visiting occasionally some inland lakes.

In conclusion, our study sheds light on the composition of the planktonic bacterial diversity in one of the most isolated regions of the Earth, particularly when environmental gradients are present, relative to the global distribution and diversity of prokaryotes. Recent studies also highlight the role of environmental variation for the distribution patterns of microorganisms in Antarctic low-nutrient environments, but they are focused in microbial mats (Valdespino-Castillo et al., 2018). All these findings should contribute to increase the comprehensive database on the prokaryotic diversity in the Antarctic continent. These data from Antarctic environments can be used for the monitoring of the effects of global warming in these remote aquatic ecosystems.

## Author Contributions

AC, AQ, and CR conceived this work. JV and DV performed the sample collection and the filtration. JM-L and AP carried out the sequence analyses. AP, CR, and AC wrote the manuscript. All authors read and approved the final manuscript.

## Conflict of Interest Statement

The authors declare that the research was conducted in the absence of any commercial or financial relationships that could be construed as a potential conflict of interest.
