# Supplementary material for: Bacterioplankton Community Composition Along Environmental Gradients in Lakes From Byers Peninsula (Maritime Antarctica) as Determined by Next-Generation Sequencing
Source: Front Microbiol. 2019 Apr 30;10:908. doi: 10.3389/fmicb.2019.00908 (PMC6503055; doi:10.3389/fmicb.2019.00908)
Supplement: Supplementary file 1 [file Data_Sheet_1.ZIP › Chester_D.html]

Javascript must be enabled to view this page.

magnitude

 2000

 1995.94

 277.7

 234.7

 174.48

 174.48

 56.08

 0

 0

 0

 39.06

 32.23

 0

 6.83

 0

 6.72

 0

 0

 21.16

 21.16

 21.16

 0

 0

 0

 0

 26.91

 26.91

 16.09

 0

 0

 16.09

 16.09

 16.09

 0

 0

 0

 0

 0

 0

 0

 0

 0

 0

 0

 54.61

 42.47

 36.38

 36.38

 36.38

 0

 0

 0

 0

 0

 0

 0

 0

 0

 0

 0

 0

 0

 0

 0

 0

 0

 0

 6.09

 12.14

 12.14

 12.14

 0

 0

 0

 0

 0

 0

 0

 0

 0

 0

 0

 1097.19

 337.05

 118.72

 118.72

 37.22

 0

 0

 0

 94.77

 0

 0

 0

 0

 0

 0

 0

 0

 0

 0

 0

 0

 0

 94.77

 0

 94.77

 0

 0

 0

 0

 33.86

 33.86

 33.86

 0

 0

 72.8

 72.8

 .12

 0

 72.68

 0

 0

 10.39

 10.39

 0

 0

 10.39

 0

 0

 0

 0

 0

 0

 0

 6.51

 6.51

 0

 0

 0

 484.03

 0

 188.92

 157.15

 .45

 0

 23.57

 0

 0

 62.41

 .13

 37.51

 0

 6.15

 0

 0

 0

 0

 0

 0

 0

 27.09

 27.09

 .14

 .14

 0

 0

 0

 0

 0

 0

 0

 4.54

 4.54

 0

 0

 0

 0

 0

 0

 0

 0

 0

 0

 0

 0

 0

 0

 48.24

 38.81

 38.81

 9.43

 9.43

 0

 0

 6.21

 5.91

 5.91

 .3

 0

 .3

 64.91

 64.91

 64.91

 0

 0

 0

 0

 0

 0

 0

 0

 0

 0

 0

 0

 0

 0

 0

 0

 0

 0

 0

 71.03

 71.03

 71.03

 0

 0

 0

 0

 0

 0

 0

 0

 0

 0

 36.88

 0

 36.88

 36.88

 .14

 .14

 .14

 0

 0

 0

 0

 0

 0

 0

 0

 0

 0

 0

 0

 0

 0

 0

 0

 0

 0

 0

 0

 0

 0

 0

 0

 0

 0

 1.7

 276.11

 231.72

 77.39

 22.41

 154.33

 0

 60.34

 0

 0

 0

 0

 0

 0

 0

 0

 0

 0

 0

 0

 0

 0

 0

 24.05

 22.4

 9.27

 0

 0

 0

 9.27

 9.27

 0

 0

 0

 0

 11.07

 0

 0

 0

 0

 0

 0

 0

 0

 0

 0

 0

 0

 0

 0

 12.41

 12.41

 12.41

 12.41

 12.41

 31.39

 31.39

 31.39

 31.39

 27.83

 0

 0

 3.56

 0

 0

 0

 0

 0

 0

 0

 0

 0

 0

 0

 0

 0

 0

 0

 0

 0

 0

 0

 0

 0

 371.49

 272.13

 171.14

 25.97

 87.27

 87.27

 0

 0

 0

 0

 57.9

 57.9

 0

 0

 0

 77.74

 0

 11.36

 0

 11.36

 0

 0

 66.38

 0

 66.38

 23.25

 22.84

 22.84

 0

 0

 0

 .2

 .2

 .21

 0

 0

 0

 0

 0

 0

 0

 0

 0

 0

 0

 0

 0

 0

 0

 0

 0

 0

 0

 0

 0

 99.36

 62.15

 62.15

 59.36

 2.79

 37.21

 37.21

 37.21

 0

 0

 0

 0

 0

 0

 0

 0

 0

 0

 0

 0

 0

 0

 0

 0

 0

 0

 0

 0

 0

 0

 0

 0

 0

 0

 0

 0

 0

 0

 0

 0

 0

 0

 0

 40.65

 40.65

 40.65

 40.65

 0

 40.65

 0

 0

 0

 0

 0

 0

 0

 0

 52.58

 52.58

 1.32

 0

 0

 1.32

 1.32

 0

 0

 0

 0

 0

 0

 42.87

 42.87

 0

 0

 8.39

 0

 0

 8.39

 8.39

 0

 0

 0

 0

 0

 0

 0

 0

 0

 0

 0

 0

 0

 0

 0

 0

 0

 0

 0

 0

 0

 0

 0

 0

 0

 10.27

 10.27

 10.27

 10.27

 10.27

 0

 0

 0

 0

 0

 0

 0

 0

 0

 0

 0

 0

 6.06

 36.68

 36.68

 0

 0

 0

 0

 0

 2.48

 0

 0

 2.48

 2.48

 0

 0

 0

 0

 0

 0

 0

 0

 0

 0

 0

 0

 0

 0

 0

 0

 0

 0

 0

 0

 0

 0

 0

 0

 0

 0

 0

 0

 0

 0

 0

 0

 0

 2.43

 2.43

 2.43

 2.43

 2.43

 0

 0

 0

 0

 0

 0

 0

 4.06

 0

 0

 0

 0

 1.95

 1.95

 0

 0

 2.11

 2.11

 2.11

 0

 0

 0

 2.11

 2.11

 0

 0
