# Supplementary material for: Bacterioplankton Community Composition Along Environmental Gradients in Lakes From Byers Peninsula (Maritime Antarctica) as Determined by Next-Generation Sequencing
Source: Front Microbiol. 2019 Apr 30;10:908. doi: 10.3389/fmicb.2019.00908 (PMC6503055; doi:10.3389/fmicb.2019.00908)
Supplement: Supplementary file 1 [file Data_Sheet_1.ZIP › Chester_S.html]

Javascript must be enabled to view this page.

magnitude

 2000

 1956.55

 415.4

 413.38

 351.93

 350.29

 170.81

 0

 0

 1.64

 36.97

 0

 0

 36.97

 0

 36.97

 0

 0

 24.48

 0

 0

 24.48

 24.48

 0

 0

 0

 0

 2.02

 0

 0

 2.02

 2.02

 0

 2.02

 0

 0

 0

 0

 0

 0

 0

 0

 0

 0

 18.45

 18.45

 18.45

 0

 0

 0

 0

 0

 0

 0

 0

 0

 18.45

 18.45

 0

 0

 0

 0

 0

 0

 0

 0

 0

 0

 0

 0

 0

 0

 0

 0

 0

 0

 0

 0

 0

 0

 0

 0

 1042.22

 77.11

 13.22

 13.22

 .15

 0

 0

 0

 42.42

 0

 0

 24.87

 24.87

 0

 7.71

 0

 7.71

 0

 9.84

 0

 9.84

 0

 0

 0

 0

 0

 0

 0

 0

 0

 0

 0

 0

 0

 21.47

 21.47

 17.81

 0

 0

 0

 3.66

 0

 0

 0

 0

 0

 0

 0

 0

 0

 0

 0

 0

 0

 0

 0

 0

 0

 934.61

 0

 684.75

 564.8

 0

 0

 0

 0

 70.26

 274.16

 22.39

 7.56

 0

 143.24

 .4

 0

 .16

 0

 0

 .17

 0

 0

 0

 66.19

 66.19

 0

 0

 0

 0

 0

 0

 0

 48.72

 48.72

 0

 0

 0

 0

 0

 0

 0

 0

 0

 44.31

 44.31

 44.31

 0

 0

 0

 0

 0

 0

 0

 0

 0

 22.42

 .1

 .1

 22.32

 13.96

 8.36

 26.58

 0

 0

 14.93

 14.93

 0

 11.65

 11.65

 140.2

 140.2

 140.2

 .11

 .11

 0

 .11

 0

 0

 0

 0

 0

 0

 0

 9.47

 9.47

 9.47

 0

 0

 0

 0

 0

 0

 0

 0

 0

 0

 0

 0

 0

 0

 1.4

 1.4

 1.4

 0

 0

 0

 0

 0

 0

 0

 0

 0

 0

 0

 0

 0

 0

 0

 0

 0

 0

 0

 0

 0

 0

 0

 0

 0

 0

 0

 30.5

 4.79

 0

 0

 4.79

 0

 0

 0

 0

 0

 0

 0

 0

 0

 0

 0

 0

 0

 0

 0

 0

 0

 0

 0

 0

 0

 0

 0

 0

 0

 0

 0

 0

 0

 0

 0

 0

 0

 0

 19.34

 0

 0

 0

 0

 0

 0

 0

 0

 0

 0

 0

 0

 0

 0

 0

 0

 0

 0

 0

 0

 0

 0

 0

 0

 0

 0

 0

 0

 0

 0

 0

 0

 0

 0

 0

 0

 0

 0

 0

 0

 0

 0

 0

 232.98

 216.35

 8.22

 0

 0

 0

 0

 8.22

 0

 0

 0

 0

 0

 0

 0

 79.95

 0

 79.95

 29.38

 50.57

 0

 0

 0

 0

 0

 89.5

 34.36

 34.36

 0

 12.13

 12.13

 .27

 .27

 42.74

 0

 0

 0

 0

 0

 0

 38.68

 38.68

 38.68

 0

 0

 0

 0

 0

 0

 0

 0

 0

 0

 0

 0

 0

 0

 0

 0

 0

 0

 0

 0

 16.63

 0

 16.63

 16.63

 0

 0

 0

 0

 46.83

 0

 0

 0

 0

 0

 0

 0

 0

 0

 0

 0

 0

 0

 0

 0

 0

 0

 0

 0

 0

 0

 0

 0

 46.83

 46.83

 0

 78.07

 78.07

 78.07

 78.07

 44.26

 33.81

 0

 0

 0

 0

 22.88

 22.88

 22.88

 22.88

 74.54

 74.54

 9.67

 0

 0

 9.67

 9.67

 0

 0

 0

 0

 0

 0

 55.67

 55.67

 0

 0

 0

 0

 0

 0

 0

 8.78

 8.78

 8.78

 0

 0

 0

 0

 0

 0

 0

 11.92

 11.92

 11.92

 0

 0

 0

 11.92

 11.92

 0

 0

 0

 0

 0

 0

 0

 0

 0

 0

 0

 0

 0

 0

 0

 0

 0

 0

 0

 0

 0

 0

 0

 0

 0

 0

 0

 0

 0

 0

 0

 0

 13.26

 13.26

 13.26

 0

 0

 0

 0

 0

 0

 0

 0

 0

 0

 0

 0

 0

 0

 0

 0

 0

 0

 0

 0

 0

 0

 0

 0

 0

 0

 0

 0

 0

 0

 0

 0

 0

 0

 0

 0

 0

 0

 0

 0

 0

 0

 0

 0

 0

 0

 0

 43.45

 0

 0

 0

 0

 43.45

 0

 43.45

 43.45

 0

 0

 0

 0

 0

 0

 0

 0

 0

 0
