# Supplementary material for: Bacterioplankton Community Composition Along Environmental Gradients in Lakes From Byers Peninsula (Maritime Antarctica) as Determined by Next-Generation Sequencing
Source: Front Microbiol. 2019 Apr 30;10:908. doi: 10.3389/fmicb.2019.00908 (PMC6503055; doi:10.3389/fmicb.2019.00908)
Supplement: Supplementary file 1 [file Data_Sheet_1.ZIP › Escondido_S.html]

Javascript must be enabled to view this page.

magnitude

 2000

 1967.24

 301.74

 226.5

 122.69

 115.65

 49.71

 7.04

 7.04

 0

 103.81

 21.4

 4.35

 82.41

 19.41

 2.51

 0

 0

 0

 0

 0

 0

 0

 0

 0

 8.3

 8.3

 25.01

 1.38

 1.38

 0

 0

 0

 0

 0

 23.63

 41.93

 28.1

 28.1

 13.83

 6.93

 6.9

 6.9

 0

 203.89

 128.18

 121.39

 .09

 .09

 0

 0

 75.32

 66.81

 8.51

 20.73

 20.73

 0

 0

 0

 0

 0

 0

 25.25

 25.25

 6.79

 6.79

 6.79

 0

 64.21

 64.21

 16.85

 16.85

 22.96

 22.96

 0

 0

 0

 0

 11.5

 11.5

 11.5

 11.5

 787.15

 290.44

 18.25

 18.25

 .14

 0

 2.86

 0

 111.05

 21.14

 21.14

 0

 0

 0

 26.23

 21.08

 5.15

 0

 17.88

 17.69

 0

 .19

 18.44

 18.44

 0

 0

 0

 27.36

 27.36

 32.67

 0

 0

 32.67

 0

 77.87

 77.87

 0

 30.33

 17.63

 6.55

 17.99

 20.12

 20.12

 11.21

 8.91

 0

 0

 0

 0

 0

 30.48

 30.48

 30.48

 0

 0

 0

 0

 0

 456.35

 0

 187.62

 184.74

 .12

 25.72

 0

 98.09

 12.52

 6.26

 .25

 21.89

 0

 2.84

 0

 4.05

 0

 0

 0

 0

 0

 0

 0

 2.88

 2.88

 0

 0

 0

 0

 0

 0

 0

 0

 0

 0

 0

 0

 0

 0

 20.66

 20.66

 20.66

 0

 1.03

 1.03

 1.03

 0

 0

 3.34

 0

 0

 3.34

 3.34

 0

 0

 64.72

 59.68

 59.68

 5.04

 0

 5.04

 75.43

 42.47

 42.47

 32.96

 0

 32.96

 0

 0

 0

 0

 0

 41.02

 41.02

 28.05

 0

 0

 0

 0

 0

 21.85

 21.85

 21.85

 16.87

 16.87

 2.69

 0

 0

 0

 0

 0

 0

 0

 0

 0

 0

 14.73

 14.73

 0

 0

 .8

 .8

 .8

 7.28

 0

 0

 7.28

 7.28

 0

 0

 0

 0

 0

 0

 0

 0

 0

 0

 0

 0

 0

 0

 0

 0

 0

 0

 0

 0

 1

 0

 40.36

 0

 0

 0

 0

 0

 0

 0

 21.95

 0

 0

 0

 12.67

 12.67

 0

 9.28

 9.28

 0

 0

 0

 0

 0

 0

 0

 2.49

 2.49

 2.49

 0

 0

 0

 0

 15.24

 15.24

 15.24

 .68

 .68

 .68

 0

 0

 0

 0

 0

 0

 0

 0

 0

 0

 0

 0

 67.2

 67.2

 67.2

 67.2

 67.2

 24.01

 24.01

 0

 0

 0

 0

 0

 0

 24.01

 24.01

 21.31

 2.7

 0

 0

 0

 0

 0

 0

 0

 0

 0

 0

 0

 0

 0

 0

 0

 0

 0

 201.01

 177.36

 8.01

 0

 0

 0

 0

 0

 8.01

 8.01

 0

 0

 0

 0

 0

 52.47

 24.88

 27.59

 0

 27.59

 0

 0

 0

 0

 0

 41.13

 41.13

 41.13

 0

 0

 0

 0

 0

 0

 54.89

 2.13

 28.08

 28.08

 24.68

 0

 20.86

 20.86

 12.25

 8.61

 0

 0

 0

 0

 0

 0

 0

 0

 0

 0

 0

 19.01

 8.08

 8.08

 0

 8.08

 10.93

 10.93

 10.93

 4.64

 0

 4.64

 4.64

 0

 0

 0

 0

 42.98

 25.02

 25.02

 25.02

 0

 12.99

 12.99

 12.99

 12.99

 0

 .11

 0

 0

 0

 0

 0

 0

 0

 0

 4.86

 4.86

 4.86

 4.86

 0

 0

 0

 0

 0

 0

 0

 0

 0

 0

 0

 0

 0

 0

 8.64

 8.64

 8.64

 8.64

 208.32

 208.32

 101.16

 101.16

 101.16

 0

 0

 0

 0

 0

 32.81

 32.81

 10.92

 29.96

 29.96

 0

 37.82

 3.4

 3.4

 0

 0

 0

 0

 0

 0

 55.93

 55.93

 40.7

 11.83

 15.23

 15.23

 15.23

 26.22

 26.22

 15.23

 15.23

 13.62

 1.61

 0

 0

 10.99

 10.99

 10.99

 0

 0

 0

 0

 14.48

 14.48

 14.39

 14.39

 14.39

 0

 0

 0

 0

 0

 0

 0

 .09

 0

 0

 0

 0

 0

 25.67

 0

 25.67

 25.67

 25.67

 25.67

 0

 0

 0

 0

 0

 0

 0

 0

 0

 0

 0

 0

 0

 0

 0

 0

 0

 0

 0

 0

 0

 0

 0

 0

 0

 0

 0

 0

 0

 0

 0

 0

 0

 0

 0

 0

 0

 0

 0

 0

 0

 0

 0

 0

 0

 0

 0

 0

 0

 0

 0

 32.76

 18

 18

 18

 18

 0

 0

 0

 0

 14.76

 14.76

 14.76

 14.76

 6.69

 8.07

 0

 0

 0

 0

 0
