# Supplementary material for: Bacterioplankton Community Composition Along Environmental Gradients in Lakes From Byers Peninsula (Maritime Antarctica) as Determined by Next-Generation Sequencing
Source: Front Microbiol. 2019 Apr 30;10:908. doi: 10.3389/fmicb.2019.00908 (PMC6503055; doi:10.3389/fmicb.2019.00908)
Supplement: Supplementary file 1 [file Data_Sheet_1.ZIP › Limnopolar_D.html]

Javascript must be enabled to view this page.

magnitude

 2000

 2000

 38.03

 1.98

 0

 0

 0

 0

 0

 0

 1.98

 0

 0

 1.98

 0

 0

 0

 0

 0

 0

 0

 0

 0

 0

 0

 0

 0

 36.05

 36.05

 36.05

 0

 0

 0

 0

 0

 0

 0

 0

 0

 0

 0

 0

 0

 0

 163.42

 16.49

 16.49

 .97

 .97

 0

 0

 0

 0

 0

 0

 0

 0

 0

 0

 0

 0

 0

 15.52

 15.52

 0

 0

 0

 0

 146.93

 146.93

 0

 0

 146.93

 146.93

 0

 0

 0

 0

 0

 0

 0

 0

 1325.61

 279.97

 .96

 .96

 0

 0

 0

 .96

 254.77

 0

 0

 0

 0

 0

 254.77

 254.77

 0

 0

 0

 0

 0

 0

 0

 0

 0

 0

 0

 0

 0

 0

 0

 0

 0

 0

 24.24

 24.24

 .94

 0

 23.3

 0

 0

 0

 0

 0

 0

 0

 0

 0

 0

 0

 0

 0

 0

 0

 0

 0

 0

 0

 906.4

 0

 275.38

 218.89

 0

 0

 0

 25.4

 24.2

 157.5

 0

 0

 0

 3.95

 0

 0

 0

 0

 0

 .96

 0

 0

 0

 0

 0

 0

 56.49

 56.49

 0

 0

 0

 0

 0

 0

 0

 0

 0

 0

 0

 0

 0

 0

 0

 412.18

 412.18

 412.18

 0

 0

 0

 0

 0

 0

 0

 0

 0

 35.85

 30

 30

 5.85

 0

 5.85

 0

 0

 0

 0

 0

 0

 0

 0

 48.46

 48.46

 48.46

 0

 0

 0

 0

 0

 0

 0

 0

 0

 0

 0

 0

 0

 0

 0

 0

 0

 0

 0

 0

 0

 0

 0

 0

 0

 0

 0

 0

 60.22

 60.22

 60.22

 0

 0

 0

 0

 0

 72.37

 0

 0

 0

 0

 0

 0

 0

 0

 0

 0

 0

 0

 0

 0

 0

 0

 0

 0

 0

 0

 0

 139.24

 108.05

 0

 0

 108.05

 0

 0

 0

 0

 0

 0

 0

 0

 0

 0

 0

 0

 0

 0

 0

 0

 0

 0

 0

 30.22

 29.23

 0

 0

 .99

 .99

 0

 .97

 .97

 .97

 0

 0

 0

 0

 0

 0

 0

 0

 0

 0

 0

 0

 0

 0

 0

 0

 0

 0

 0

 0

 0

 0

 0

 0

 0

 0

 0

 0

 0

 0

 0

 0

 0

 0

 0

 0

 0

 0

 0

 0

 0

 0

 0

 0

 0

 0

 0

 0

 0

 56.26

 23.29

 0

 0

 0

 0

 0

 0

 0

 0

 0

 0

 0

 0

 0

 0

 0

 0

 0

 0

 0

 0

 0

 0

 0

 23.29

 23.29

 23.29

 0

 0

 0

 0

 0

 0

 0

 0

 0

 0

 0

 0

 0

 0

 0

 0

 0

 0

 0

 0

 0

 0

 0

 0

 0

 0

 0

 0

 0

 0

 0

 0

 0

 0

 0

 32.97

 0

 32.97

 0

 32.97

 32.97

 0

 0

 6.81

 0

 0

 0

 0

 0

 0

 0

 0

 0

 0

 0

 0

 0

 0

 0

 0

 0

 0

 0

 0

 0

 0

 0

 6.81

 6.81

 0

 0

 0

 0

 0

 0

 0

 .96

 0

 0

 0

 0

 0

 0

 0

 51.53

 51.53

 0

 0

 0

 0

 0

 0

 0

 0

 51.53

 51.53

 0

 0

 0

 0

 0

 0

 0

 0

 0

 0

 0

 0

 0

 0

 0

 0

 0

 0

 0

 0

 124.05

 124.05

 65.9

 0

 0

 0

 65.9

 65.9

 0

 0

 0

 58.15

 58.15

 58.15

 0

 233.33

 232.37

 145.94

 145.94

 144.95

 0

 0

 0

 0

 0

 0

 0

 54.41

 32.02

 0

 .96

 0

 0

 0

 0

 0

 0

 0

 0

 0

 0

 0

 0

 0

 0

 0

 0

 0

 0

 0

 0

 0

 0

 0

 0

 0

 0

 0

 0

 0

 0

 0

 0

 0

 0

 0

 0

 0

 0

 0

 0

 0

 0

 0

 0

 0

 0

 0

 0

 0

 0

 0

 0

 0

 0

 0

 0

 0

 0

 0

 0

 0

 0

 0

 0

 0

 0

 0

 0

 0

 0

 0

 0

 0

 0

 0

 0

 0

 0

 0
