# Supplementary material for: Bacterioplankton Community Composition Along Environmental Gradients in Lakes From Byers Peninsula (Maritime Antarctica) as Determined by Next-Generation Sequencing
Source: Front Microbiol. 2019 Apr 30;10:908. doi: 10.3389/fmicb.2019.00908 (PMC6503055; doi:10.3389/fmicb.2019.00908)
Supplement: Supplementary file 1 [file Data_Sheet_1.ZIP › Limnopolar_S.html]

Javascript must be enabled to view this page.

magnitude

 2000

 1889.18

 122.21

 122.21

 108.56

 78.78

 0

 0

 0

 29.78

 13.65

 11.05

 11.05

 2.6

 0

 0

 0

 0

 0

 0

 0

 0

 0

 0

 0

 0

 0

 0

 0

 0

 0

 0

 0

 0

 0

 0

 0

 0

 0

 0

 0

 0

 0

 0

 66.62

 9.52

 9.52

 0

 0

 0

 0

 0

 0

 0

 0

 0

 0

 0

 0

 9.52

 0

 9.52

 0

 0

 0

 0

 0

 0

 57.1

 57.1

 0

 0

 0

 0

 57.1

 57.1

 0

 0

 0

 0

 0

 0

 1003.29

 38.68

 21.1

 21.1

 0

 0

 0

 .57

 10.12

 0

 0

 9.63

 0

 9.63

 0

 0

 0

 0

 .49

 .49

 0

 0

 0

 0

 0

 0

 0

 0

 0

 0

 0

 0

 0

 0

 7.46

 7.46

 0

 0

 7.46

 0

 0

 0

 0

 0

 0

 0

 0

 0

 0

 0

 0

 0

 0

 0

 0

 0

 0

 0

 893.04

 0

 350.61

 331.7

 0

 0

 0

 0

 61.3

 156.72

 15.12

 2.23

 51.42

 31.9

 0

 0

 0

 13.01

 0

 0

 0

 0

 0

 .53

 .53

 0

 18.38

 18.38

 0

 0

 0

 0

 0

 0

 0

 0

 0

 0

 0

 22.88

 22.88

 0

 0

 3.62

 3.62

 3.62

 0

 0

 0

 0

 0

 0

 0

 0

 0

 196.75

 170.79

 170.79

 25.96

 0

 25.96

 0

 0

 0

 0

 0

 0

 0

 0

 89.57

 89.57

 89.57

 .53

 .53

 0

 0

 0

 0

 0

 0

 0

 0

 0

 203.58

 203.58

 203.58

 0

 0

 0

 0

 0

 0

 0

 0

 0

 0

 0

 0

 0

 0

 0

 0

 0

 21.88

 21.88

 21.88

 0

 0

 0

 0

 0

 0

 0

 0

 3.16

 3.16

 3.16

 0

 0

 0

 0

 0

 0

 0

 0

 0

 0

 0

 0

 .46

 71.57

 0

 0

 0

 0

 0

 0

 0

 0

 0

 0

 0

 0

 0

 0

 0

 0

 0

 0

 0

 0

 0

 0

 0

 68.91

 68.91

 0

 6.9

 0

 0

 0

 2.66

 2.66

 2.66

 0

 0

 0

 0

 0

 0

 0

 0

 0

 0

 0

 0

 0

 0

 0

 0

 0

 0

 0

 0

 54.38

 54.38

 35.63

 35.63

 0

 19.91

 15.72

 0

 0

 0

 0

 0

 18.75

 18.75

 0

 0

 0

 0

 0

 0

 0

 0

 0

 0

 0

 0

 0

 0

 0

 128.7

 127.64

 12.56

 0

 0

 0

 0

 7.84

 0

 0

 0

 0

 4.72

 4.72

 0

 0

 0

 0

 0

 0

 0

 0

 0

 0

 0

 93.04

 68.79

 68.79

 0

 0

 0

 24.25

 24.25

 0

 13.45

 10.92

 0

 0

 2.53

 0

 8.59

 8.59

 8.59

 0

 0

 0

 0

 0

 0

 0

 0

 0

 0

 0

 0

 0

 0

 0

 0

 0

 0

 0

 0

 1.06

 0

 0

 0

 0

 0

 1.06

 1.06

 0

 0

 0

 0

 0

 0

 0

 0

 0

 0

 0

 0

 0

 0

 0

 0

 0

 0

 0

 0

 0

 0

 0

 0

 0

 0

 0

 0

 0

 0

 0

 0

 0

 0

 0

 0

 0

 28.56

 28.56

 28.56

 28.56

 137.83

 137.83

 92.03

 0

 0

 92.03

 92.03

 0

 0

 0

 0

 0

 0

 20.84

 20.84

 0

 0

 24.96

 24.96

 0

 0

 0

 0

 0

 0

 0

 0

 0

 0

 0

 0

 0

 76.11

 76.11

 0

 0

 0

 0

 0

 0

 76.11

 76.11

 76.11

 0

 0

 0

 0

 99.19

 84.57

 84.57

 84.57

 44.96

 0

 29.69

 9.92

 0

 0

 0

 0

 0

 0

 0

 14.62

 0

 0

 0

 0

 0

 0

 0

 0

 0

 56.44

 11.36

 0

 45.08

 0

 0

 0

 0

 0

 0

 0

 0

 0

 0

 0

 41.8

 41.8

 41.8

 44.99

 37.26

 37.26

 37.26

 37.26

 7.73

 7.73

 7.73

 0

 7.73

 0

 0

 0

 0

 0

 0

 0

 0

 0

 0

 0

 0

 0

 0

 0

 0

 0

 0

 0

 0

 0

 0

 91.88

 0

 0

 0

 0

 91.88

 91.88

 0

 0

 0

 0

 0

 0

 0

 0

 0

 0

 0

 0

 18.94
