# Supplementary material for: Bacterioplankton Community Composition Along Environmental Gradients in Lakes From Byers Peninsula (Maritime Antarctica) as Determined by Next-Generation Sequencing
Source: Front Microbiol. 2019 Apr 30;10:908. doi: 10.3389/fmicb.2019.00908 (PMC6503055; doi:10.3389/fmicb.2019.00908)
Supplement: Supplementary file 1 [file Data_Sheet_1.ZIP › Midge_S.html]

Javascript must be enabled to view this page.

magnitude

 2000

 1977.41

 376.61

 328.11

 321.14

 321.14

 65.08

 0

 0

 0

 1.27

 .53

 .53

 .74

 0

 0

 0

 0

 5.7

 .26

 .26

 0

 0

 5.44

 5.44

 18.03

 18.03

 9

 0

 0

 9

 9

 0

 9

 0

 0

 21.47

 21.47

 21.47

 0

 0

 0

 0

 0

 127.96

 123.22

 123.22

 123.22

 0

 32.49

 46.99

 0

 0

 0

 0

 0

 0

 0

 0

 0

 0

 0

 0

 0

 0

 0

 0

 0

 4.74

 4.74

 0

 0

 0

 0

 0

 0

 4.74

 4.74

 0

 0

 0

 0

 836.87

 18.4

 0

 0

 0

 0

 0

 0

 .58

 0

 0

 .29

 0

 .29

 0

 0

 0

 0

 0

 0

 0

 0

 .29

 0

 .29

 0

 0

 0

 0

 0

 0

 0

 0

 0

 17.82

 17.82

 0

 0

 17.82

 0

 0

 0

 0

 0

 0

 0

 0

 0

 0

 0

 0

 0

 0

 0

 0

 0

 0

 0

 759.55

 0

 311.96

 300.17

 .11

 108.79

 0

 0

 8.32

 10.75

 .36

 76

 0

 64.53

 0

 26.15

 0

 0

 2.38

 0

 0

 0

 0

 2.99

 0

 2.99

 0

 0

 0

 0

 0

 0

 0

 0

 0

 8.8

 8.8

 0

 0

 0

 0

 0

 0

 4.56

 4.56

 4.56

 0

 0

 0

 0

 0

 0

 0

 0

 0

 1.62

 1.49

 1.49

 .13

 0

 .13

 3.63

 1.42

 1.42

 0

 0

 0

 2.21

 2.21

 218.63

 218.63

 218.63

 0

 0

 0

 0

 43.63

 43.63

 19.03

 0

 0

 0

 0

 125.89

 125.89

 125.89

 0

 0

 0

 0

 0

 0

 0

 0

 0

 0

 0

 0

 0

 0

 .12

 .12

 .12

 0

 0

 0

 0

 0

 0

 0

 0

 0

 0

 0

 20.05

 20.05

 20.05

 0

 0

 0

 0

 0

 0

 0

 0

 0

 0

 0

 0

 27.05

 40.89

 25.35

 0

 0

 25.35

 12.84

 0

 0

 3.53

 0

 0

 0

 0

 0

 0

 0

 0

 0

 0

 0

 0

 0

 0

 0

 8.2

 0

 0

 0

 8.2

 0

 8.2

 0

 0

 0

 3.56

 3.56

 3.56

 0

 0

 0

 .13

 0

 .13

 .13

 0

 0

 0

 0

 0

 0

 0

 0

 0

 0

 57.15

 9.39

 9.39

 9.39

 9.39

 0

 0

 0

 0

 0

 0

 0

 0

 0

 0

 0

 0

 0

 32.12

 32.12

 18.82

 13.3

 13.3

 0

 0

 0

 0

 15.64

 0

 231.83

 228.85

 6.26

 0

 0

 0

 0

 6.26

 0

 0

 0

 0

 0

 0

 0

 117.34

 0

 117.34

 94.08

 23.26

 0

 0

 0

 0

 0

 87.67

 56.84

 56.84

 0

 30.83

 30.83

 0

 0

 0

 9.29

 0

 0

 0

 9.29

 0

 8.29

 8.29

 5.74

 0

 2.55

 0

 0

 0

 0

 0

 0

 0

 0

 0

 0

 0

 0

 0

 0

 0

 0

 0

 0

 2.98

 0

 0

 0

 0

 0

 2.98

 2.98

 27.51

 0

 0

 0

 24.67

 0

 0

 0

 0

 0

 0

 0

 0

 0

 0

 0

 0

 0

 0

 0

 0

 0

 0

 0

 1.88

 1.88

 .96

 8.26

 8.26

 8.26

 8.26

 5.6

 2.66

 0

 0

 0

 0

 10.56

 10.56

 10.56

 10.56

 140.83

 140.83

 .75

 0

 0

 .75

 .75

 0

 0

 0

 0

 0

 0

 31.89

 31.89

 0

 54.43

 11.57

 11.57

 0

 0

 0

 38.98

 38.98

 38.98

 0

 0

 0

 0

 0

 0

 0

 .94

 .94

 .94

 0

 0

 0

 .94

 .94

 0

 0

 0

 0

 0

 0

 0

 71.9

 71.9

 59.49

 59.49

 35

 0

 0

 0

 0

 0

 0

 7.41

 0

 5

 0

 0

 0

 11.87

 0

 0

 0

 0

 0

 0

 0

 5.74

 0

 0

 0

 0

 5.74

 5.74

 13.61

 13.61

 13.61

 13.61

 16.36

 16.36

 16.36

 16.36

 0

 0

 0

 0

 0

 0

 0

 0

 0

 0

 0

 0

 0

 0

 0

 0

 0

 0

 39.41

 0

 0

 0

 0

 0

 0

 0

 0

 0

 0

 0

 0

 0

 0

 0

 0

 0

 0

 0

 0

 0

 0

 0

 0

 0

 0

 0

 0

 0

 0

 0

 0

 0

 0

 0

 22.59
