# Supplementary material for: Bacterioplankton Community Composition Along Environmental Gradients in Lakes From Byers Peninsula (Maritime Antarctica) as Determined by Next-Generation Sequencing
Source: Front Microbiol. 2019 Apr 30;10:908. doi: 10.3389/fmicb.2019.00908 (PMC6503055; doi:10.3389/fmicb.2019.00908)
Supplement: Supplementary file 1 [file Data_Sheet_1.ZIP › Refugio_S.html]

Javascript must be enabled to view this page.

magnitude

 2000

 1993.36

 332.66

 217.19

 139.97

 139.97

 38.8

 0

 0

 0

 43.5

 .38

 .38

 43.12

 11.82

 11.98

 3.17

 33.72

 0

 0

 0

 0

 0

 0

 0

 .62

 .62

 103.46

 91.2

 91.2

 12.26

 9.53

 .12

 9.41

 2.73

 0

 9.34

 9.34

 2.54

 0

 0

 0

 0

 2.05

 16.14

 14.62

 14.62

 0

 0

 0

 0

 7.12

 0

 0

 0

 0

 0

 0

 0

 7.5

 5.99

 1.51

 0

 0

 0

 0

 0

 0

 0

 0

 0

 0

 0

 0

 0

 0

 0

 0

 1.52

 1.52

 1.52

 1.52

 1048.92

 112.77

 8.86

 8.86

 0

 1.68

 0

 1.81

 28.42

 7.26

 7.26

 3.71

 0

 3.71

 5.02

 .54

 0

 1.53

 .09

 0

 .09

 0

 4.46

 0

 4.46

 5.74

 5.74

 2.14

 2.14

 3.94

 2.42

 2.42

 1.52

 1.52

 44.73

 44.73

 7.55

 15.78

 11.21

 0

 0

 0

 0

 0

 0

 0

 5.41

 5.41

 5.41

 4.11

 0

 0

 0

 0

 0

 3.54

 3.54

 3.54

 749.6

 36.94

 351.72

 176.58

 2.19

 3.52

 0

 .29

 0

 75.65

 8.87

 26.64

 0

 2.75

 15.19

 8.76

 1.73

 0

 0

 1.52

 2.52

 .14

 .14

 15.09

 15.09

 0

 51.89

 39.97

 11.92

 46.4

 42.62

 3.78

 23.91

 8.14

 5.05

 14.62

 14.62

 13.96

 9.13

 25.29

 25.29

 0

 25.29

 63.06

 29.02

 29.02

 34.04

 34.04

 2.27

 0

 0

 0

 0

 2.27

 2.27

 1.32

 .21

 .21

 1.11

 0

 1.11

 0

 0

 0

 0

 0

 0

 0

 0

 9.35

 9.35

 9.35

 0

 0

 0

 0

 .13

 .13

 .13

 47.58

 0

 0

 0

 20.42

 15.14

 1.39

 13.75

 5.28

 5.28

 67.86

 50.08

 47.32

 17.06

 5.94

 2.76

 2.76

 0

 0

 0

 0

 .89

 .89

 .89

 2.43

 0

 0

 2.43

 0

 .93

 18.63

 4.53

 4.53

 4.53

 8.75

 5.51

 5.51

 5.51

 3.75

 1.83

 1.83

 1.83

 1.14

 1.14

 1.14

 2.39

 1.58

 1.58

 1.58

 1.82

 2.32

 186.55

 14.91

 4.44

 4.44

 10.47

 2.44

 3.19

 2.12

 90.93

 27.55

 24.3

 3.25

 0

 0

 11.18

 0

 0

 31.09

 11.1

 2.33

 2.33

 2.32

 0

 0

 3.7

 3.7

 3.7

 0

 0

 0

 0

 5.85

 5.85

 5.85

 32.75

 13.44

 13.44

 4.38

 4.38

 0

 7.68

 4.89

 2.79

 0

 7.91

 9.17

 2.53

 2.53

 2.53

 0

 0

 0

 0

 0

 36.29

 8.83

 2.86

 2.86

 0

 0

 0

 0

 0

 0

 0

 0

 0

 0

 0

 5.97

 5.97

 5.97

 10.95

 8.89

 0

 8.89

 0

 4.48

 2.68

 1.73

 2.06

 0

 16.51

 235.01

 232.62

 29.64

 5.82

 4.73

 0

 4.73

 5.21

 0

 0

 6.26

 6.26

 4.78

 4.78

 2.84

 30.24

 0

 8.16

 4.12

 0

 0

 4.04

 22.08

 20.87

 0

 47.18

 43.81

 41.98

 0

 3.37

 3.37

 0

 0

 0

 46.55

 32.77

 0

 0

 3.96

 9.82

 79.01

 37.87

 37.87

 0

 0

 8.07

 8.07

 5.63

 5.63

 14.06

 7.29

 4.91

 6.6

 1.93

 4.67

 0

 0

 0

 0

 0

 0

 0

 0

 2.39

 0

 2.39

 2.39

 0

 0

 0

 0

 133.07

 0

 0

 0

 7.63

 31.22

 31.22

 31.22

 24.28

 6.94

 53.27

 7.79

 7.79

 7.79

 12.7

 10.34

 10.34

 7.2

 2.36

 0

 0

 0

 0

 2.58

 17.62

 17.62

 8.05

 19.56

 19.56

 19.56

 19.56

 13.48

 6.08

 21.42

 14.46

 14.46

 14.46

 0

 0

 0

 0

 64.5

 64.5

 33.21

 0

 0

 23.57

 19.7

 6.37

 3.27

 3.27

 0

 0

 0

 29.36

 29.36

 2.02

 0

 1.93

 1.93

 1.93

 0

 0

 0

 0

 0

 0

 0

 0

 0

 0

 0

 0

 2.14

 0

 0

 0

 0

 0

 0

 0

 0

 0

 0

 0

 0

 0

 2.14

 42.07

 32.49

 17.24

 17.24

 3.18

 2.65

 1.99

 0

 12.73

 12.73

 12.73

 2.52

 0

 0

 3.52

 4.21

 1.85

 0

 11

 9.33

 1.52

 1.52

 1.52

 1.52

 .15

 0

 0

 0

 0

 0

 0

 0

 0

 0

 0

 0

 0

 0

 0

 0

 0

 0

 0

 6.07

 0

 0

 0

 0

 6.07

 6.07

 6.07

 2.02

 0

 13.35

 8.95

 7.08

 1.87

 4.43

 3.81

 3.81

 3.81

 3.81

 3.81

 0

 0

 0

 0

 0

 1.56

 1.56

 1.56

 1.36

 1.36

 1.36

 1.36

 6.64

 0

 0

 0

 0

 4.58

 4.58

 0

 0

 2.06

 0

 0

 0

 0

 0

 0

 0

 2.06

 2.06

 0
