# Supplementary material for: Bacterioplankton Community Composition Along Environmental Gradients in Lakes From Byers Peninsula (Maritime Antarctica) as Determined by Next-Generation Sequencing
Source: Front Microbiol. 2019 Apr 30;10:908. doi: 10.3389/fmicb.2019.00908 (PMC6503055; doi:10.3389/fmicb.2019.00908)
Supplement: Supplementary file 1 [file Data_Sheet_1.ZIP › Somero_S.html]

Javascript must be enabled to view this page.

magnitude

 2000

 2000

 107.82

 38.28

 3.1

 3.1

 0

 0

 0

 0

 35.18

 28.8

 0

 6.38

 5.14

 0

 0

 0

 0

 0

 0

 0

 0

 0

 0

 17.42

 17.42

 0

 0

 0

 0

 0

 0

 0

 0

 0

 52.12

 0

 0

 52.12

 52.12

 0

 0

 0

 0

 0

 0

 0

 0

 0

 0

 0

 0

 0

 0

 0

 0

 0

 0

 0

 0

 0

 0

 0

 0

 0

 0

 0

 0

 0

 0

 0

 0

 0

 0

 0

 0

 0

 0

 0

 0

 0

 765.48

 72.8

 .48

 .48

 0

 0

 0

 0

 6.88

 0

 0

 0

 0

 0

 0

 0

 0

 0

 6.88

 0

 0

 6.88

 0

 0

 0

 0

 0

 0

 0

 0

 0

 0

 0

 0

 65.44

 65.44

 0

 0

 0

 0

 65.44

 0

 0

 0

 0

 0

 0

 0

 0

 0

 0

 0

 0

 0

 0

 0

 0

 0

 620.47

 0

 304.79

 302.14

 0

 42

 0

 0

 0

 41.51

 0

 199.32

 0

 .54

 0

 18.34

 0

 0

 0

 0

 0

 0

 0

 0

 0

 0

 2.65

 2.65

 0

 0

 0

 0

 0

 0

 0

 0

 0

 0

 0

 0

 0

 0

 0

 0

 0

 0

 0

 0

 0

 0

 0

 0

 0

 0

 0

 8.66

 8.66

 8.66

 0

 0

 0

 0

 0

 0

 0

 0

 0

 0

 0

 166.62

 166.62

 166.62

 14.17

 14.17

 0

 14.17

 0

 0

 0

 0

 0

 0

 0

 60.29

 60.29

 60.29

 0

 0

 0

 0

 0

 0

 0

 0

 0

 0

 0

 0

 0

 0

 1.7

 1.7

 1.7

 0

 0

 0

 0

 0

 0

 0

 0

 0

 0

 0

 0

 0

 0

 0

 0

 0

 0

 0

 0

 0

 0

 0

 0

 0

 0

 19.23

 72.21

 .48

 0

 0

 .48

 0

 0

 0

 0

 0

 0

 0

 0

 0

 0

 0

 0

 0

 0

 0

 0

 0

 0

 0

 0

 0

 0

 0

 0

 0

 0

 71.73

 71.73

 71.73

 0

 0

 0

 0

 0

 0

 0

 0

 0

 0

 0

 0

 0

 0

 0

 0

 0

 0

 0

 0

 13.16

 0

 0

 0

 0

 0

 0

 0

 0

 0

 0

 0

 0

 0

 0

 0

 0

 0

 0

 0

 0

 0

 0

 0

 0

 0

 0

 0

 13.16

 942.3

 885.52

 0

 0

 0

 0

 0

 0

 0

 0

 0

 0

 0

 0

 0

 0

 0

 0

 0

 0

 0

 0

 0

 0

 0

 818.37

 818.37

 818.37

 0

 0

 0

 0

 0

 0

 0

 0

 0

 0

 0

 0

 67.15

 67.15

 67.15

 0

 0

 0

 0

 0

 0

 0

 0

 0

 0

 0

 0

 0

 0

 0

 0

 0

 0

 0

 0

 56.78

 56.78

 0

 0

 0

 0

 0

 0

 .4

 0

 0

 0

 0

 0

 0

 0

 0

 0

 0

 0

 0

 0

 0

 0

 0

 0

 0

 0

 0

 0

 0

 0

 .4

 .4

 0

 0

 0

 0

 0

 0

 0

 88.34

 0

 0

 0

 0

 0

 0

 0

 0

 0

 0

 0

 0

 0

 0

 0

 0

 0

 0

 0

 0

 0

 0

 0

 0

 0

 0

 0

 0

 0

 0

 0

 0

 0

 0

 0

 0

 0

 0

 0

 0

 0

 0

 0

 0

 0

 0

 0

 0

 0

 0

 0

 0

 0

 0

 61.17

 61.17

 61.17

 61.17

 47.61

 13.56

 0

 0

 0

 0

 0

 0

 0

 0

 0

 0

 0

 0

 21.33

 0

 0

 0

 0

 0

 21.33

 0

 0

 0

 0

 0

 0

 0

 0

 0

 0

 0

 0

 0

 0

 0

 0

 0

 0

 0

 0

 0

 0

 0

 0

 0

 0

 0

 0

 0

 0

 0

 0

 0

 0

 0

 0

 0

 0

 0

 0

 0

 0

 0

 0

 0

 0

 0

 0

 0

 0

 0

 0

 0

 0

 0

 0

 0

 0

 0

 0

 0

 0

 0

 0

 0

 0

 0

 0

 0

 0
