# Supplementary material for: Bacterioplankton Community Composition Along Environmental Gradients in Lakes From Byers Peninsula (Maritime Antarctica) as Determined by Next-Generation Sequencing
Source: Front Microbiol. 2019 Apr 30;10:908. doi: 10.3389/fmicb.2019.00908 (PMC6503055; doi:10.3389/fmicb.2019.00908)
Supplement: Supplementary file 1 [file Data_Sheet_1.ZIP › Turbio_S.html]

Javascript must be enabled to view this page.

magnitude

 2000

 2000

 313.05

 287.58

 192.21

 192.21

 13.1

 0

 0

 0

 95.37

 93.68

 0

 1.69

 0

 0

 0

 0

 0

 0

 0

 0

 0

 0

 0

 0

 0

 25.47

 0

 0

 25.47

 25.47

 0

 25.47

 0

 0

 0

 0

 0

 0

 0

 0

 0

 0

 97.95

 97.95

 97.95

 0

 0

 0

 0

 0

 0

 0

 0

 0

 97.95

 0

 97.95

 0

 0

 0

 0

 0

 0

 0

 0

 0

 0

 0

 0

 0

 0

 0

 0

 0

 0

 0

 0

 0

 0

 0

 1031.48

 271.82

 223.48

 223.48

 203.56

 0

 0

 15.78

 0

 0

 0

 0

 0

 0

 0

 0

 0

 0

 0

 0

 0

 0

 0

 0

 0

 0

 0

 0

 0

 0

 0

 0

 0

 0

 48.34

 48.34

 0

 15.2

 33.14

 0

 0

 0

 0

 0

 0

 0

 0

 0

 0

 0

 0

 0

 0

 0

 0

 0

 0

 0

 722.79

 0

 402.54

 356.81

 234.1

 0

 0

 0

 .87

 55.61

 .85

 15.97

 0

 49.41

 0

 0

 0

 0

 0

 0

 0

 0

 0

 37.36

 37.36

 0

 7.56

 7.56

 0

 0

 0

 0

 .81

 .81

 0

 0

 0

 0

 0

 0

 0

 0

 0

 .84

 .84

 .84

 0

 0

 0

 0

 0

 0

 0

 0

 0

 0

 0

 0

 0

 0

 0

 0

 0

 0

 0

 0

 0

 0

 0

 176.57

 176.57

 176.57

 .8

 .8

 0

 0

 0

 0

 0

 0

 0

 0

 0

 0

 0

 0

 0

 0

 0

 0

 0

 0

 0

 0

 0

 0

 0

 0

 0

 0

 4.26

 4.26

 4.26

 0

 0

 0

 0

 0

 0

 0

 0

 0

 0

 0

 128.44

 128.44

 128.44

 0

 0

 0

 0

 0

 0

 0

 0

 0

 0

 0

 0

 0

 36.87

 0

 0

 0

 0

 0

 0

 0

 0

 0

 0

 0

 0

 0

 0

 0

 0

 0

 0

 0

 0

 0

 0

 0

 0

 0

 0

 0

 0

 0

 0

 0

 0

 0

 0

 0

 0

 0

 0

 0

 36.87

 0

 36.87

 36.87

 0

 0

 0

 0

 0

 0

 0

 0

 0

 0

 26.91

 26.91

 0

 0

 0

 0

 0

 0

 0

 0

 0

 0

 26.91

 26.91

 26.91

 0

 0

 0

 0

 0

 0

 0

 0

 0

 0

 0

 0

 0

 0

 443.63

 436.68

 21.03

 0

 0

 0

 0

 0

 0

 0

 0

 0

 21.03

 21.03

 0

 219.04

 0

 219.04

 145.11

 0

 73.93

 0

 0

 0

 0

 122.08

 116.14

 0

 116.14

 0

 0

 5.94

 5.94

 0

 34.81

 12.06

 0

 0

 22.75

 0

 39.72

 39.72

 38.91

 0

 .81

 0

 0

 0

 0

 0

 0

 0

 0

 0

 0

 0

 0

 0

 0

 0

 0

 0

 0

 6.95

 0

 6.95

 0

 6.95

 6.95

 0

 0

 0

 0

 0

 0

 0

 0

 0

 0

 0

 0

 0

 0

 0

 0

 0

 0

 0

 0

 0

 0

 0

 0

 0

 0

 0

 0

 0

 86.11

 86.11

 86.11

 86.11

 86.11

 0

 0

 0

 0

 0

 0

 0

 0

 0

 0

 0

 0

 0

 0

 0

 0

 0

 0

 0

 0

 0

 0

 0

 0

 0

 0

 0

 0

 0

 0

 0

 0

 0

 0

 0

 0

 0

 0

 0

 0

 0

 0

 0

 0

 0

 0

 0

 0

 0

 0

 0

 0

 0

 0

 0

 0

 0

 0

 0

 0

 0

 0

 0

 0

 0

 0

 0

 0

 0

 0

 0

 0

 0

 0

 0

 0

 0

 0

 0

 0

 0

 .87

 0

 0

 .87

 0

 0

 0

 0

 0

 0

 0

 0

 0

 0

 0

 0

 0

 0

 0

 0

 0

 0

 0

 0

 0

 0

 0

 0

 0

 0

 0

 0

 0

 0

 0

 0

 0

 0

 0

 0

 0

 0

 0

 0

 0

 0

 0

 0

 0

 0

 0

 0

 0

 0

 0

 0

 0

 0

 0

 0

 0

 0

 0

 0

 0

 0

 0

 0

 0

 0
