# Supplementary material for: Bacterioplankton Community Composition Along Environmental Gradients in Lakes From Byers Peninsula (Maritime Antarctica) as Determined by Next-Generation Sequencing
Source: Front Microbiol. 2019 Apr 30;10:908. doi: 10.3389/fmicb.2019.00908 (PMC6503055; doi:10.3389/fmicb.2019.00908)
Supplement: Supplementary file 2 [file Data_Sheet_2.docx]

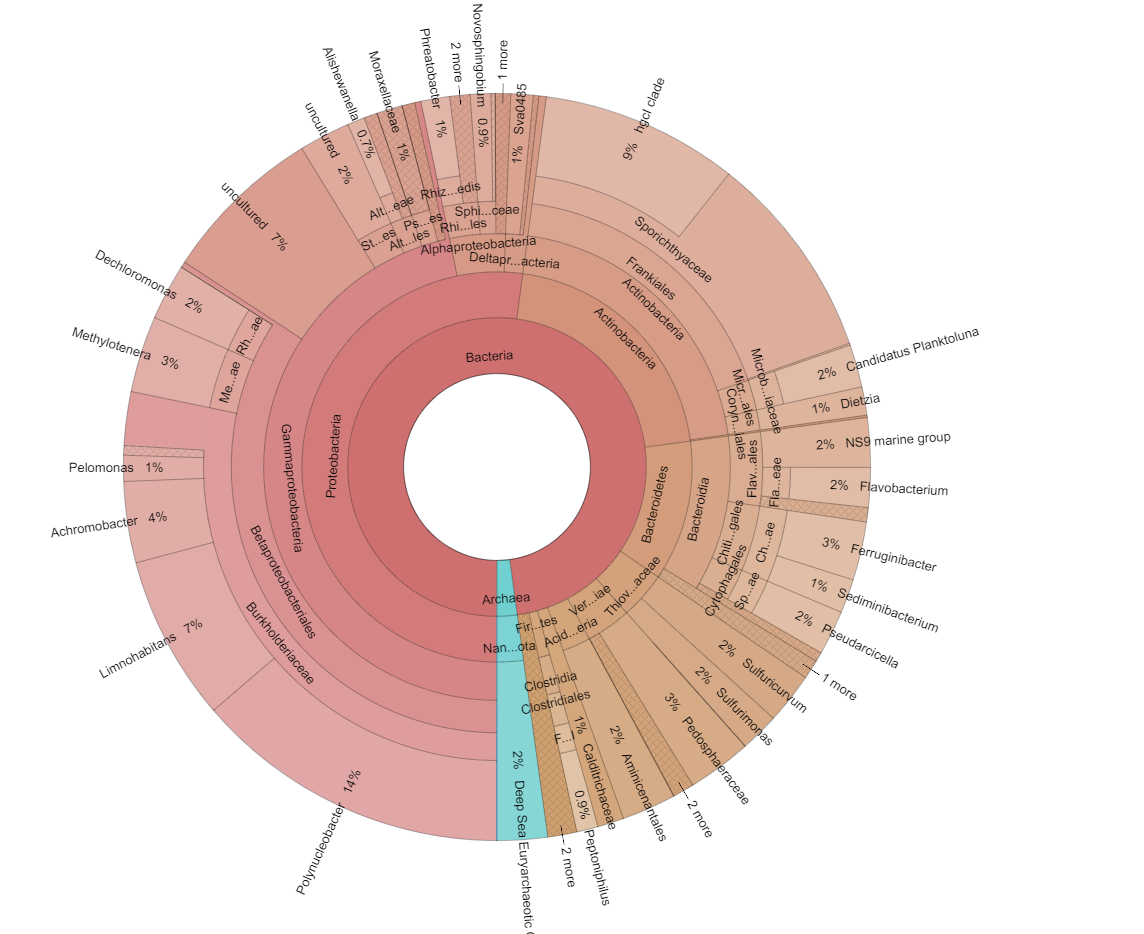


**Figure S1.** A) Graphic representation (Krona) of the taxonomic assignment of Bacteria and Archaea domains in surface water of Chester Cone Lake. Interactive link: (add link to supplementary interactive Krona chart html).


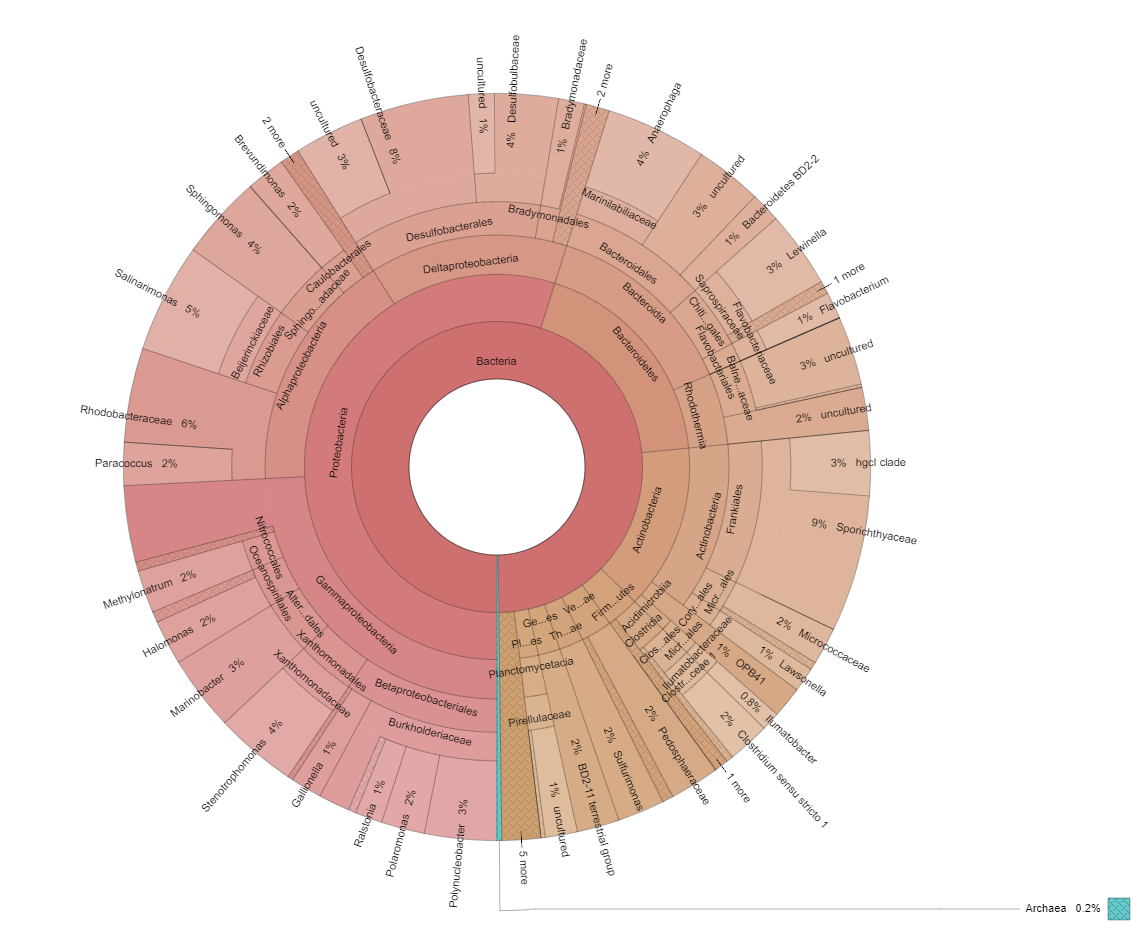


**Figure S1.** B) Graphic representation (Krona) of the taxonomic assignment of Bacteria and Archaea domains in deep water of Chester Cone Lake. Interactive link: (add link to supplementary interactive Krona chart html).


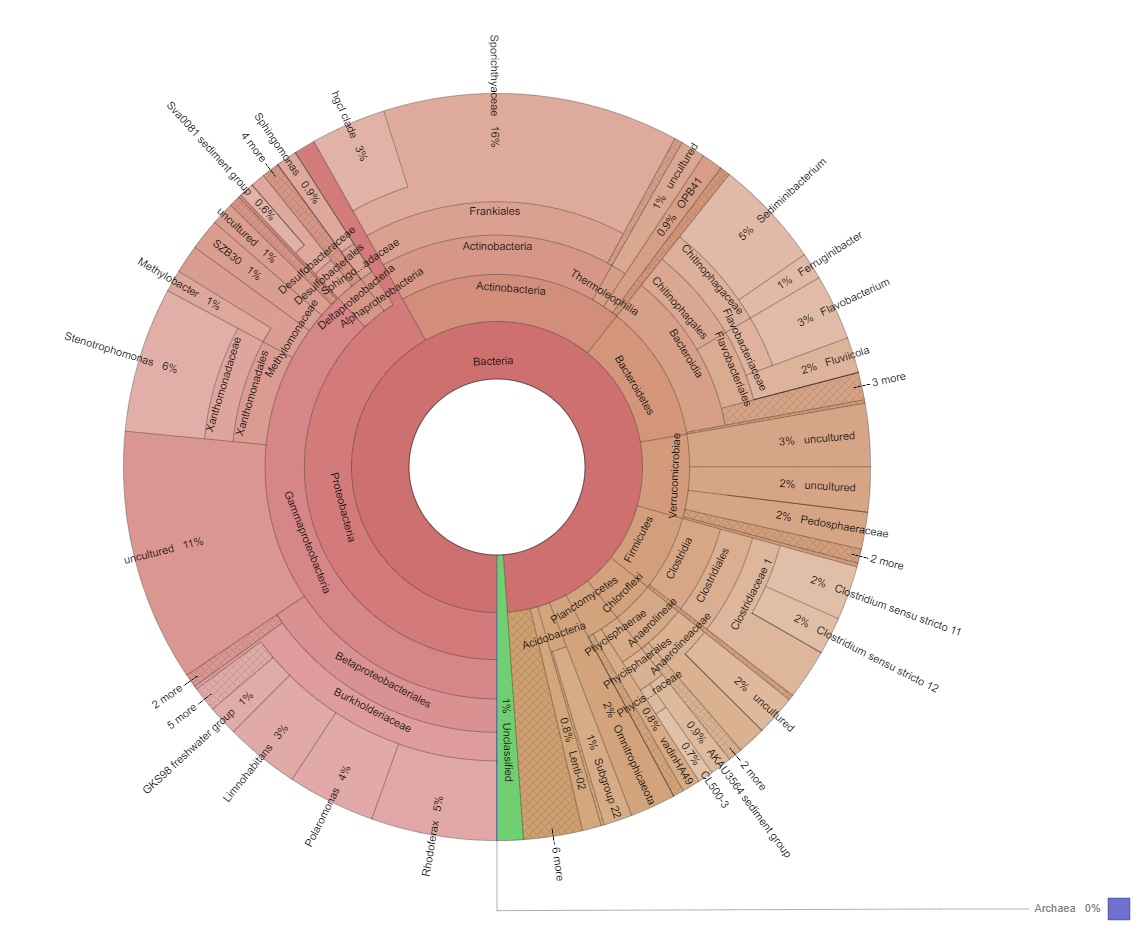


**Figure S1.** C) Graphic representation (Krona) of the taxonomic assignment of Bacteria and Archaea domains in surface water of Midge Lake. Interactive link: (add link to supplementary interactive Krona chart html).


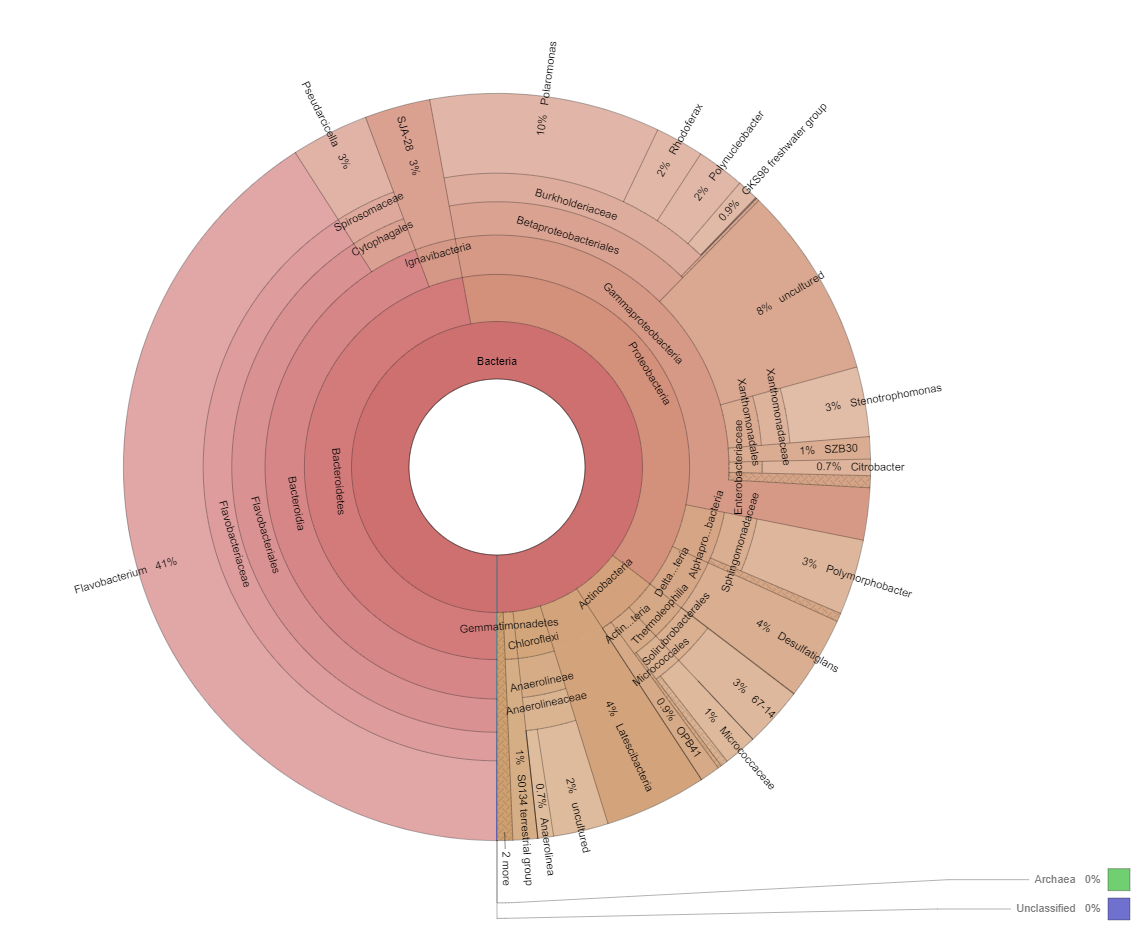


**Figure S1.** D) Graphic representation (Krona) of the taxonomic assignment of Bacteria and Archaea domains in surface water of Somero Lake. Interactive link: (add link to supplementary interactive Krona chart html).


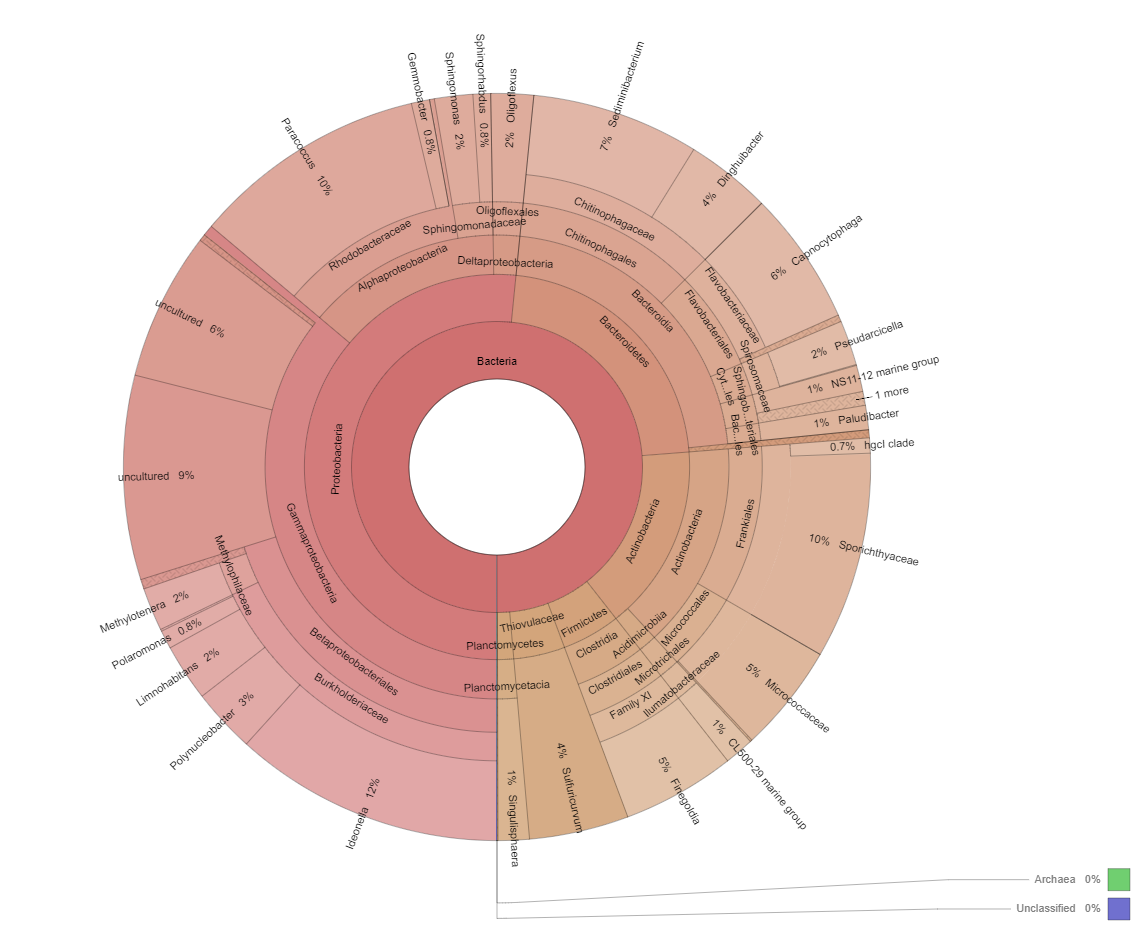


**Figure S1.** E) Graphic representation (Krona) of the taxonomic assignment of Bacteria and Archaea domains in surface water of Turbio Lake. Interactive link: (add link to supplementary interactive Krona chart html).


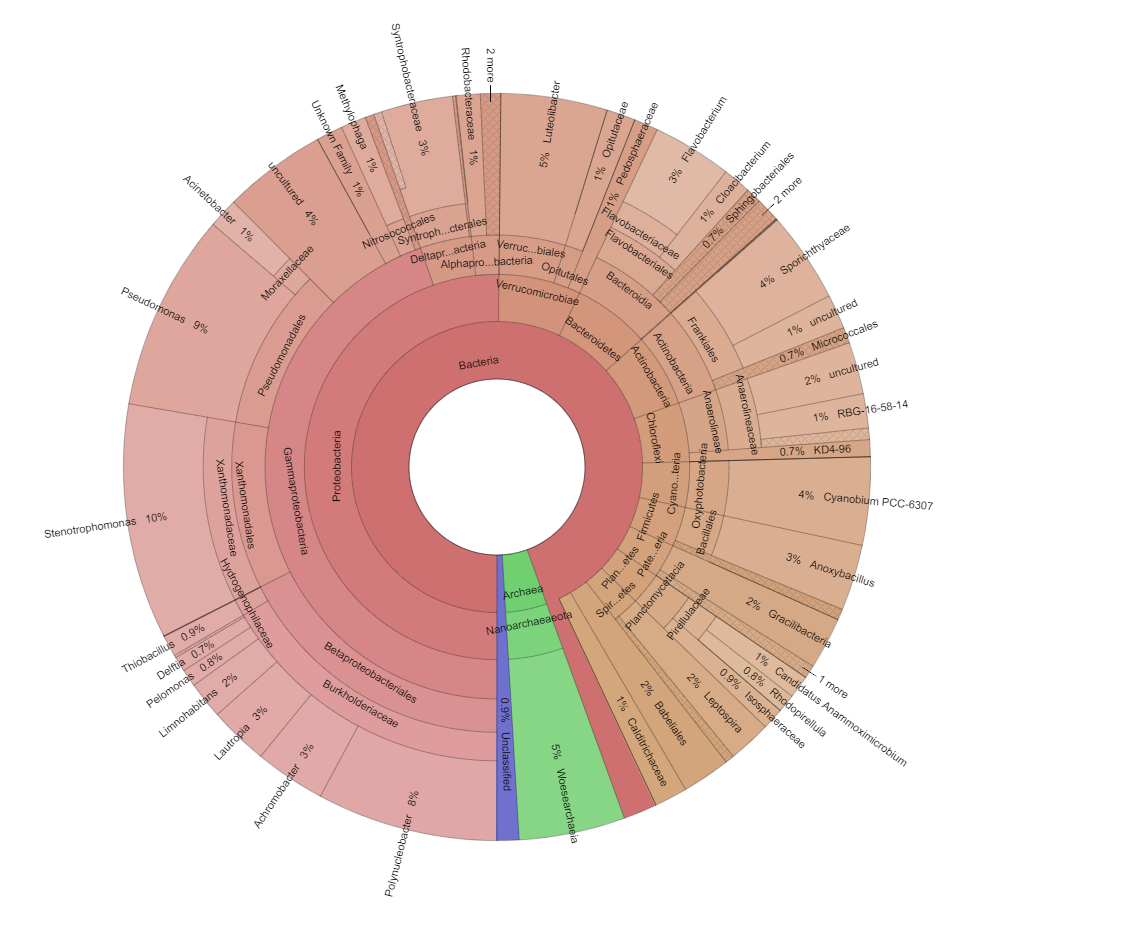


**Figure S1.** F) Graphic representation (Krona) of the taxonomic assignment of Bacteria and Archaea domains in surface water of Limnopolar Lake. Interactive link: (add link to supplementary interactive Krona chart html).


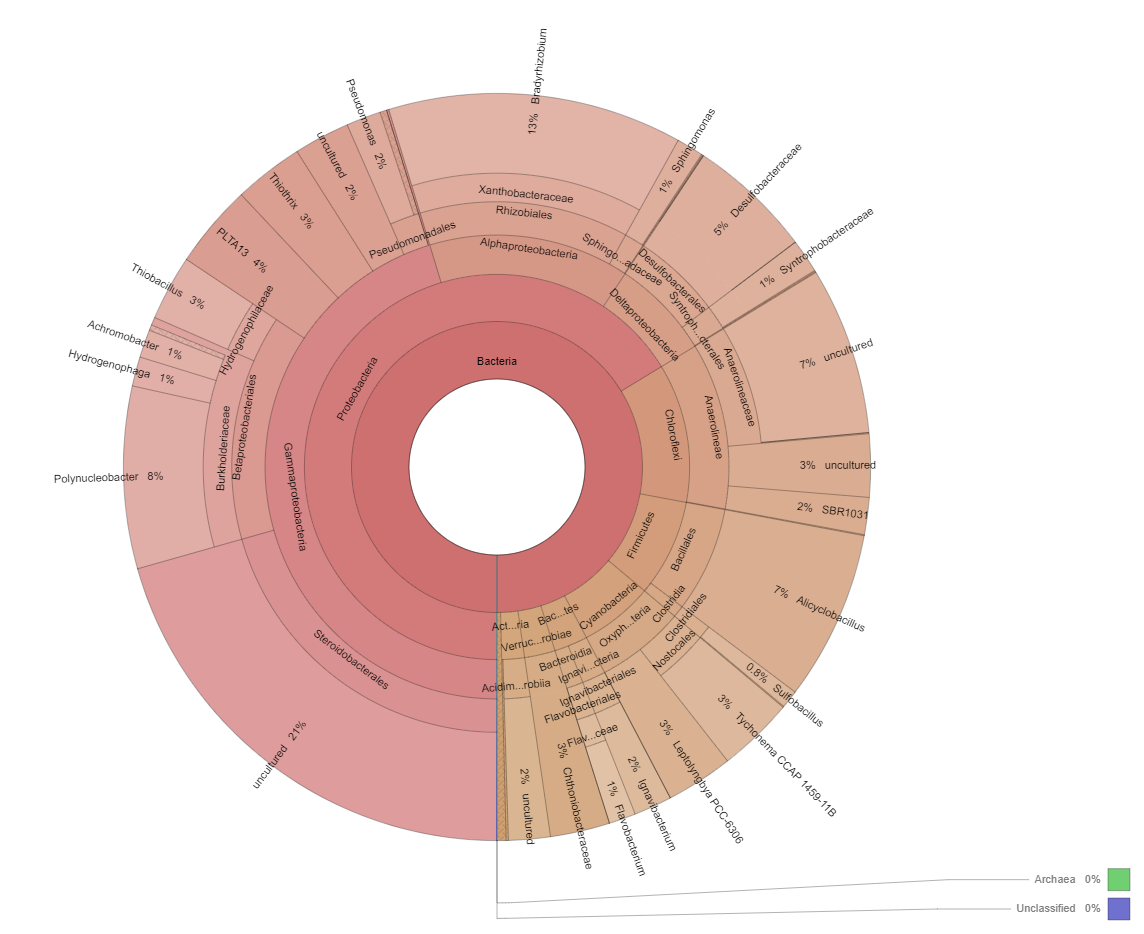


**Figure S1.** G) Graphic representation (Krona) of the taxonomic assignment of Bacteria and Archaea domains in deep water of Limnopolar Lake. Interactive link: (add link to supplementary interactive Krona chart html).


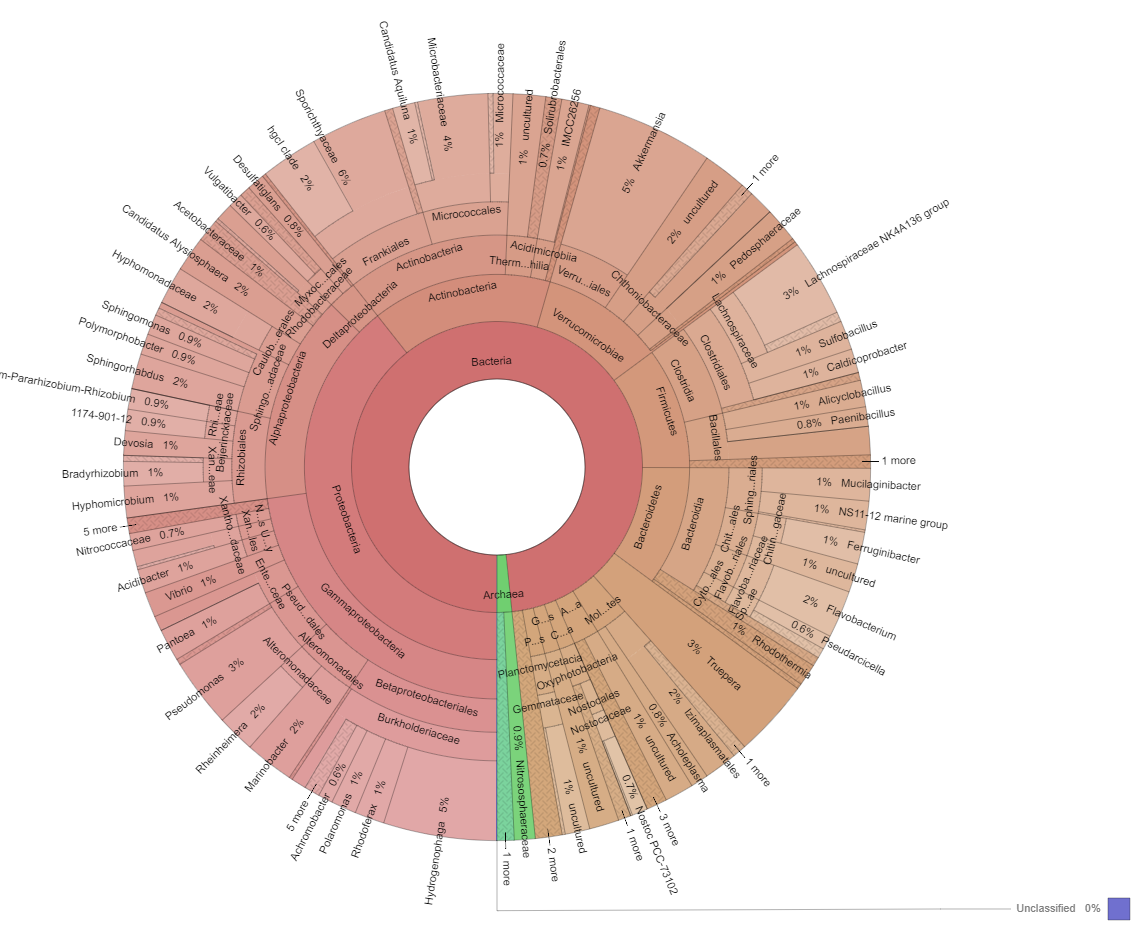


**Figure S1.** H) Graphic representation (Krona) of the taxonomic assignment of Bacteria and Archaea domains in surface water of Escondido Lake. Interactive link: (add link to supplementary interactive Krona chart html).


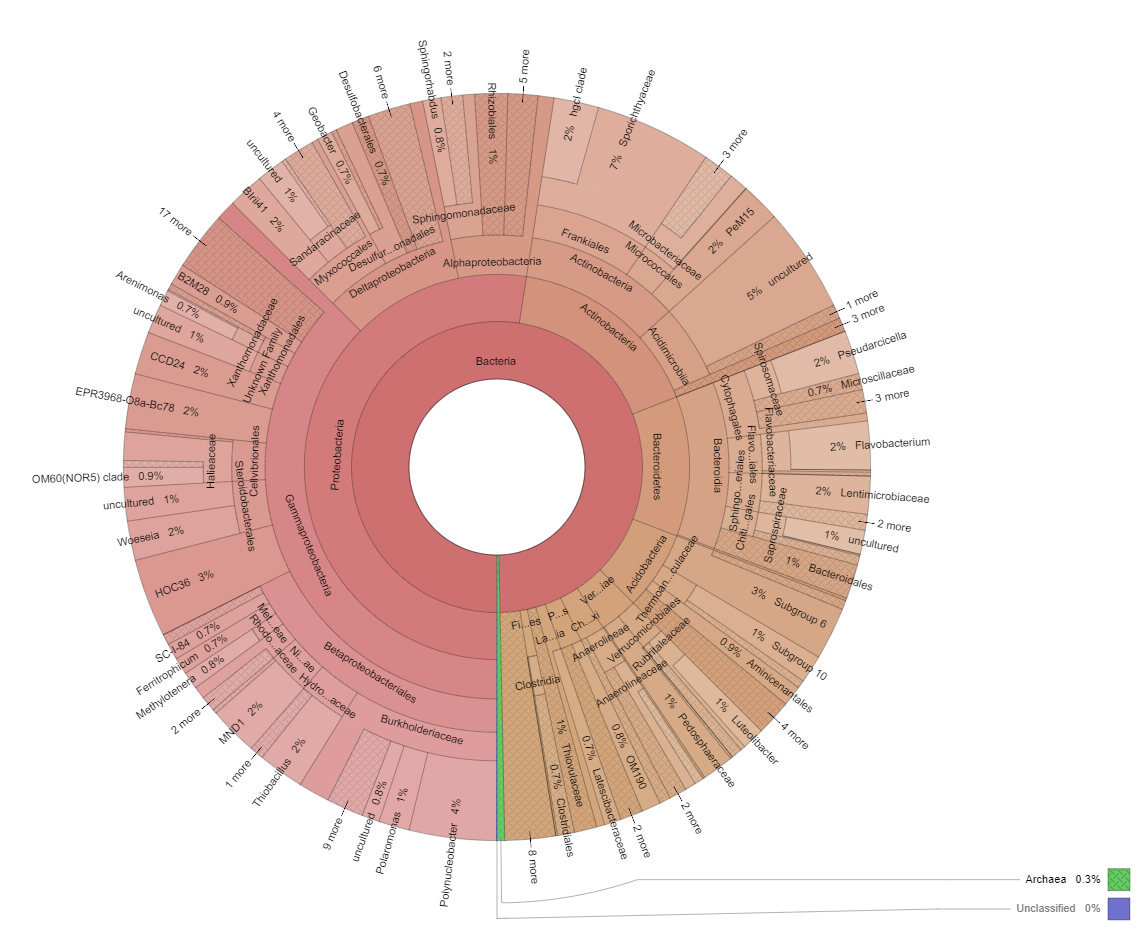


**Figure S1.** I) Graphic representation (Krona) of the taxonomic assignment of Bacteria and Archaea domains in surface water of Refugio Lake. Interactive link: (add link to supplementary interactive Krona chart html).
